# Colored Plastic Pollution Reshapes Aquatic Microbial Community and Resistance Profile via Divergent Pseudonatural Dissolved Organic Matter

**DOI:** 10.1002/advs.202514529

**Published:** 2025-10-30

**Authors:** Shuting Fang, Shuqin Liu, Jingui Huang, Yiquan Huang, Chao Chen, Qijun Ruan, Pengran Guo, Gangfeng Ouyang

**Affiliations:** ^1^ School of Chemical Engineering and Technology Sun Yat‐sen University Zhuhai 519082 China; ^2^ College of Environment and Climate Guangdong Provincial Key Laboratory of Environmental Pollution and Health Jinan University Guangzhou 511443 China; ^3^ Guangdong Provincial Key Laboratory of Chemical Measurement and Emergency Test Technology Guangdong Provincial Engineering Research Center for Ambient Mass Spectrometry Institute of Analysis, Guangdong Academy of Sciences (China National Analytical Center, Guangzhou) Guangzhou 510070 China

**Keywords:** microbial community, PE‐derived DOM heterogeneity, plastic color, resistance profile, resource availability

## Abstract

Plastic pollution alters the natural organic carbon pool in aquatic ecosystems via releasing pseudonatural dissolved organic matter (DOM). However, the composition and subsequent ecological effects of DOM leached from differently colored plastics remain unclear. Here, pronounced heterogeneity in the DOM released by colored polyethylene (PE) plastics is revealed: red PE releases higher levels of DOM, whereas white, black, and yellow PE produce more labile DOM. As a result, these labile PE‐derived DOM foster the growth of opportunistic taxa (notably copiotrophs within *Pseudomonadota*), reshaping community structure and resistance gene profiles. The elevated abundance of β‐lactamase genes highlights the potential risk of antibiotic resistance dissemination associated with black PE plastic pollution. Correlation‐based network analyses suggest that labile PE‐derived DOM inputs are associated with enhanced metabolic exchange and synergistic reallocation of available resources, accompanied by coordinated shifts in bacterial interactions and antibiotic resistance gene–host linkages. Taken together, the findings emphasize the overlooked role of plastic color in modulating DOM behavior and ecological impacts for the first time, offering targeted insights for mitigating plastic pollution and guiding sustainable production practices.

## Graphical abstract

By altering the quantity and chemical composition of polyethylene‐derived dissolved organic matter, plastic color dynamically regulates bacterial metabolic interactions, thereby reshaping community resistance expression and facilitating the dissemination of antibiotic resistance.

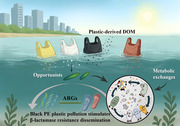

## 1 Introduction

Resource availability fundamentally shapes microbial metabolism and community dynamics in aquatic ecosystems.^[^ ^1^, ^2^, ^3^ ^]^ Thereinto, dissolved organic matter (DOM) constitutes a pivotal nutrient reservoir for metabolism of heterotrophic bacterioplankton communities.^[^ ^4^, ^5^ ^]^ It originates from aquatic and terrigenous sources and shows a complex composition covering from labile aliphatics to less reactive terrigenous phenolics.^[^ ^6^ ^]^ Different consumer communities would be associated with different DOM compounds depending on their ecological strategies for the uptake and metabolism of organic carbon.^[^ ^7^, ^8^ ^]^ Thus, the composition and availability of DOM could be a strong selection force on bacterioplankton community succession.^[^ ^9^ ^]^ Nowadays, serious environmental pollution by plastic waste is dynamically regulating the chemical composition of DOM in surrounding environments.^[^ ^10^, ^11^, ^12^, ^13^ ^]^ Plastic‐derived DOM has been becoming a non‐negligible source of organic carbon pool in aquatic ecosystems.^[^ ^14^, ^15^, ^16^ ^]^ Investigating microbial adaptations to the input of plastic‐derived DOM is essential for predicting ecosystem responses to plastic pollution. However, the understandings of the interactions between plastic pollution and microbial ecology are just beginning to emerge.^[^ ^17^, ^18^, ^19^ ^]^

Plastic‐derived DOM can serve as a significant nutritional substrate to support heterotrophic bacteria growth,^[^ ^14^, ^15^, ^20^ ^]^ although it is extremely different from natural DOM. This novel pseudonatural DOM arises from the weathering processes of plastic in the environment, particularly plastic photoaging. It consists of various additives and chain scission products, as well as unpolymerized monomers and oligomers, and accumulates at high concentrations in the water surface layer.^[^ ^21^, ^22^, ^23^ ^]^ Yet one critical aspect seems to be often overlooked: plastic colors may affect the quality and quantity of plastic‐derived DOM. Various colored plastic products are widely available in our daily life owing to different manufacturing purposes and actual usage needs. Therefore, plastic waste into the environment is colorful, not just limited to the white or transparent plastics studied in most documents. The existing studies suggest that chromatic plastics significantly affected their light absorption, thereby altering the degradation behavior of plastics.^[^ ^24^, ^25^ ^]^ Accordingly, as a bioavailable nutrient substrate, plastic‐derived DOM is expected to influence bacterial metabolism in a color‐dependent manner. This expectation rests on at least two considerations. First, there is a theoretical consequence of some levels of chemical heterogeneity and quantity variation in DOM compounds released by colored plastics, due to the changes in plastic degradation behavior. This divergence would alter the quality and quantity of bioavailable resources that plastic‐derived DOM provides to the bacterial community, thereby affecting community diversity. Second, there are some bacterial taxa display specific resource preferences.^[^ ^26^ ^]^ Color diversity increases the heterogeneity of plastic‐derived DOM, which has the potential to influence community stability and function through niche segmentation.^[^ ^27^ ^]^ In reality, the dominant color of plastic waste is distinct in different regions due to the human activities.^[^ ^11^ ^]^ With an increasing of global producer responsibility,^[^ ^28^ ^]^ elucidating the ecological impacts of colored plastic pollution will provide valuable insights for decision‐makers to optimize their production strategies.

Herein, our aim was to understand the differences of DOM released by colored plastics and quantify their bacterial associations. We employed colored polyethylene (PE) shopping bags as leaching plastics with two considerations:^[^ ^29^, ^30^ ^]^ packaging is the largest world plastics market, and PE is the most widely produced plastic type and the most abundant plastic waste worldwide. As a result, a large influx of waste PE bags should contribute most to the organic carbon pool available in aquatic environments. We systemically quantified differences in the levels and compositions of DOM released by four widely used colored PE bags. Microcosm experiments were then designed to assess their effects on bacterial communities and antibiotic resistance genes (ARGs). To further investigate the potential effects of PE‐derived DOM inputs on ecological relationships, we constructed three co‐occurrence networks: bacterial interaction network, ARG–host linkages, and bacteria–DOM associations. Our results suggested that i) white, black, and yellow PE released more labile DOM, contrasting with higher levels of DOM leached from red PE. Different PE‐derived DOM inputs ii) reshaped community structure via selective enrichment of bacterial taxa, and iii) further dynamically modulated ARG profiles. Thereinto, the facilitation of β‐lactamase resistance by black PE plastic pollution is particularly concerning. Finally, iv) correlation‐based network analyses suggest that labile PE‐derived DOM are associated with enhanced metabolic exchange and synergistic reallocation of available resources, dynamically modulating bacterial interactions and ARG–host associations. Overall, our findings reveal that plastic color governs plastic‐derived DOM characteristics and subsequent bacterial and ARG responses, highlighting the overlooked ecological implications of colored plastic pollution.

## 2 Results

### 2.1 Color‐Dependent DOM Release Heterogeneity in PE Debris

**Figure 1:**
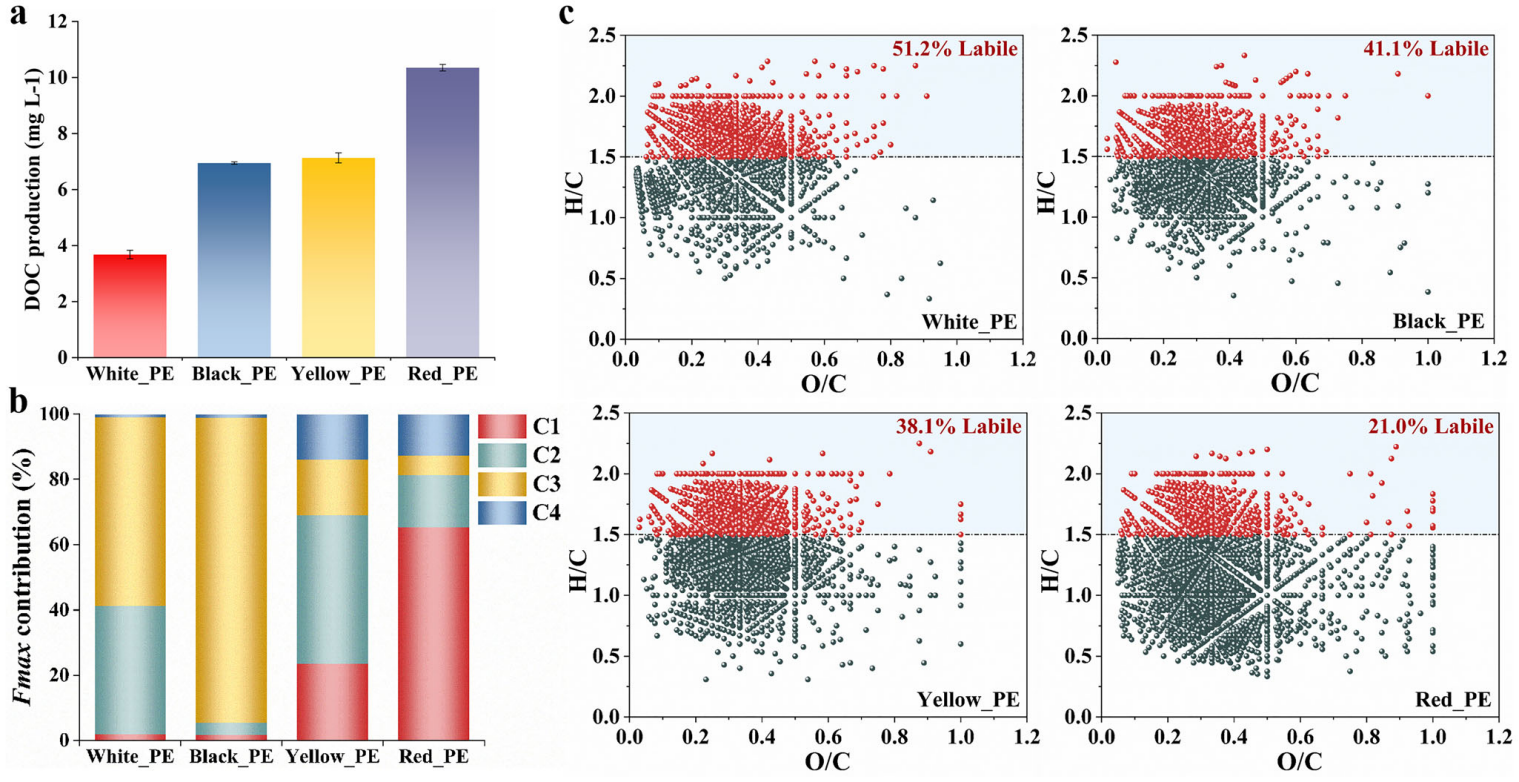
Color‐dependent DOM release heterogeneity in PE bags from molecular signatures to bioavailability gradients. a) Total DOC levels produced by each color PE debris after 14 days of exposure to simulated light radiation (*n* = 3). b) The maximum fluorescence intensities (*F*_max_) of the identified four fluorescent components were utilized to represent the relative proportions of the components in different PE‐derived DOM samples (*n* = 3). c) Van Krevelen diagrams displayed that black, white, and yellow PE released more DOM molecular formulas with a high index of lability (H/C ratio ≥ 1.5) under light radiation.

Leaching dynamics of pseudonatural DOM from differently colored PE carrier bags were distinct in three main ways. First, red PE released higher levels of dissolved organic carbon (DOC) and more abundant DOM molecules (the total number of DOM molecular formulas identified by Fourier transform ion cyclotron resonance mass spectrometry (FT‐ICR MS) analysis). After 14‐day ultraviolet irradiation, the average concentrations of the released DOM ranged from 3.98 ± 0.62 mg C/L to 10.43 ± 0.18 mg C/L following the PE color sequence of red > yellow > black > white (**Figure** **1**a). Likewise, the pattern of these PE‐derived DOM molecular richness followed that of their DOC concentrations (Figure S1, Supporting Information).

Second, protein/phenol‐like DOM compounds were mainly released by white, black, and yellow PE, contrasting with humic‐like prevalence in red PE. Four fluorescent components (C1–C4) were identified from fluorescence spectra of PE‐derived DOM via excitation–emission matrix coupled with parallel factor analysis (EEM‐PARAFAC) (Figures S2 and S3, Table S1, Supporting Information). The protein/phenol‐like (C2 + C3) components (accounted for over 95.0% of the fluorescent components on average) comprised the major DOM that was released by both white and black PE. They also remained the predominant component (62.6%) in yellow PE‐derived DOM (Figure 1b). By contrast, the humic‐like components (C1 + C4) dominated the composition of red PE‐derived DOM (78.2%), and might be attributable to polymer photolysis.^[^ ^21^ ^]^ Accordingly, CHO and CHON compounds, such as lignin‐like, and saturated compounds also comprised the majority of the molecular composition within all PE‐derived DOM (Figure S4, Supporting Information). Lots of PE‐derived DOM were characterized as lignin‐like compounds, especially for red PE‐derived DOM (comprising 74.7% of assigned molecular formulas). Meanwhile, white, black, and yellow PE also released more saturated compounds, with different proportions from 32.7% to 43.1%.

Finally, white, black, and yellow PE released more labile DOM. Of the molecular formulas detected in the leached DOM from white, black, and yellow PE (Figure 1c), 51.2%, 41.1%, and 38.1% had a high lability index (i.e., the ratios of hydrogen to carbon (H/C) ≥ 1.5), respectively. And these highly labile compounds were relatively abundant, accounted for 79.1%, 75.6%, and 80.9% of the normalized peak intensity. Despite being more abundance in molecular formulas, red PE‐derived DOM had fewer labile constituents (21.0%), and showed a higher recalcitrance.^[^ ^31^ ^]^ Higher values of O/C (the ratios of oxygen to carbon), DBE (double bond equivalents), AI_Mod_ (modified aromaticity index), and NOSC (nominal oxidation state of carbon) but a lower H/C ratio were observed in leached DOM from red PE (Table S2, Supporting Information). Given the observed high proportion of humic‐like components, red PE‐derived DOM may include more polymer degradation products. These results indicated a distinct shift in the concentration and chemical composition of DOM released by differently colored PE bags under the same light conditions.

Additionally, the aging surfaces of all colored PE plastics were assessed by scanning electron microscope (SEM) and Fourier‐transform infrared spectroscopy (FTIR). As shown in Figure S5 of the Supporting Information, SEM observations revealed that the original samples of all colors exhibited clear surface textures with smooth and uniform morphologies. But after irradiation, black PE developed ridge‐like wrinkles and a few isolated pits, whereas the yellow PE showed only shallow surface fissures. The white PE exhibited slight roughening but retained an overall intact structure. In contrast, red PE experienced the most severe surface damage, characterized by more erosion pits and partial lamellar exfoliation. FTIR analysis showed that all samples exhibited the characteristic –CH_2_ absorption bands of PE at 2915, 2850, 1470, and 717 cm^−1^ prior to aging (Figure S6, Supporting Information). In addition, black and yellow PE displayed pronounced peaks at ≈1100 and 620 cm^−1^, which are likely associated with additives or pigment‐related absorptions. After irradiation, the characteristic backbone peaks of PE (2915, 2850, 1470, and 717 cm^−1^) remained observable in all samples, indicating that the polyethylene framework was largely preserved. However, red PE exhibited the most pronounced attenuation of these backbone features, corresponding to its severe surface deterioration observed in SEM images. Notably, black and yellow PE showed significant reductions in the bands at 1110 cm^−1^ (C–O stretching) and ≈620 cm^−1^ (C–H deformation), suggesting that aging primarily resulted in the degradation or loss of additive‐ or pigment‐related components in these materials.

### 2.2 PE‐Derived DOM Inputs Foster Copiotrophs Growth and Affects Bacterial Co‐occurrence

Adding PE‐derived DOM introduced more dissolved carbons into each microcosm (1.24–2.2 mg C/L) compared to the control, as well as slightly increased concentrations of inorganic nitrogen (NO_2_^−^, NO_3_^−^, NH_4_^+^) and phosphate (PO_4_^3−^) nutrients (Figure S7, Table S3, Supporting Information). Lake bacterial communities then responded to these PE‐derived DOM inputs by scavenging the available DOC (loss of 10%–18%) and slight inorganic N and P nutrients. Correspondingly, the level of dissolved organic nitrogen (DON) in incubating systems generally increased, in consistent with previous studies.^[^ ^14^, ^15^ ^]^ Accordingly, we further quantified the community composition and diversity in each microcosm to capture the metabolic adaptation of bacterial population to each PE‐derived DOM input.

**Figure 2:**
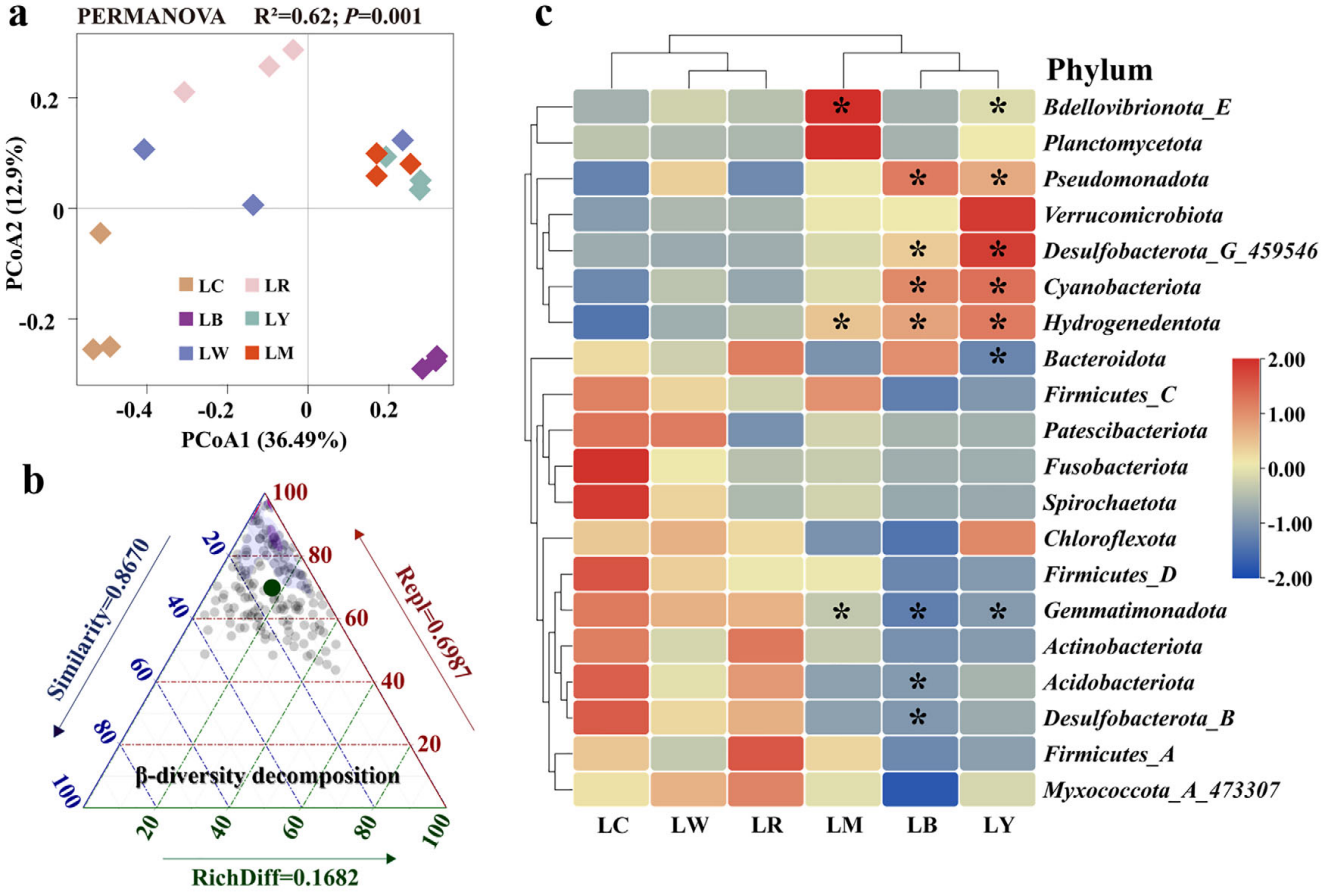
Dynamic adaptations of bacterial communities to different PE‐derived DOM inputs. a) PCoA ordination based on Bray–Curtis distances showing changes in bacterial community composition by different PE‐derived DOM supplies. Statistical analysis was performed using PERMONOVA in R. b) Triangular plots of β‐diversity comparisons (using Jaccard dissimilarity index) for bacterial communities among all samples. Each point represents a sample. Its position is determined by a triplet of values from the Similarity, Repl (replacement), and RichDiff (richness difference) matrices and the mean values of Similarity, Repl and RichDiff were shown. c) Relative abundance of the top 20 most abundant bacterial phyla among treatments via cluster heatmap, presented as the mean (*n* = 3). Asterisks indicated significant differences between the control and each PE‐derived DOM treatment (*p < 0.05, Student's *t*‐test). LC represented as the natural lake water; LB, LW, LR, and LY, respectively, expressed as the lake water added the leached DOM from black, white, red, and yellow PE. LM was set as a combined treatment, where four kinds of the collected PE‐derived DOM were converged in equal volume and acted on the same bacterial community together.

A total of 16 210 filtered sequences were retrieved across all samples based on 16S rRNA metabarcoding sequencing, which then assigned into 904 to 2545 bacterial operational taxonomic units (OTUs). After taxonomic annotation, we assessed the responses in bacterial α‐diversity to different PE‐derived DOM inputs by calculating bacterial richness (Chao 1 index) and diversity (Shannon and Simpson indexes). Although bacterial richness and diversity decreased somewhat after introducing PE‐derived DOM (except for red PE‐derived DOM), there were no discernible differences between treatments on OTU level (Figure S8, Supporting Information). Then, principal coordinate analysis (PCoA) and permutational multivariate analysis of variance (PERMANOVA) were employed to visualize and quantify the differences between bacterial community structure (β‐diversity) based on between‐sample dissimilarities (Bray–Curtis) distance (**Figure** **2**a). All PE‐derived DOM treatments formed a distinct cluster from the control along the first PCoA, and PERMANOVA confirmed the significant differences among treatments (*R* ^2^ = 0.62, *p* = 0.001, *p*‐value threshold after Bonferroni adjustment). β‐Diversity decomposition analysis subsequently showed that these significant differences were driven probably by the changes of particular bacterial taxa abundance,^[^ ^32^ ^]^ given the species replacement processes contributing 69.9% (Figure 2b).

To determine the specific effects of different PE‐derived DOM inputs on community composition, we analyzed the phylum‐ and genus‐level compositional profiles between the control and DOM‐treated samples (Figure 2c; Figure S9, Supporting Information). As shown in Figure 2c, *Pseudomonadota* comprised the dominant phylum across treatments with the largest relative abundance, and fast grew in the microcosms enriched with leached DOM from white, black, and yellow PE, as well as in PE‐derived DOM mixture treatment. *Cyanobacteriota* followed the *Pseudomonadota*, showing a similar growing tendency, while *Acidobacteriota* and *Desulfobacterota_B* precisely exhibited an opposite behavior, which growth was restricted to varying degrees. *Bdellovibrionota_E* displayed a significantly greater abundance after treated by PE‐derived DOM mixture and yellow PE‐derived DOM. *Bacteroidota* was prevalent but with a significant reduction after introducing yellow PE‐derived DOM and slight increased, along with *Firmicutes_A*, under red PE‐derived DOM input. Although there was no significant difference between treatments, a varying degree of increased abundance was noticed in *Verrucomicrobiota* across PE‐derived DOM treated samples. Interestingly, such bacterial taxon became the dominant phylum in the microcosms enriched with the black and yellow PE‐derived DOM. In the genus‐level composition (Figure S9, Supporting Information), all PE‐derived DOM treatments exhibited a marked enrichment of typical copiotrophic genera compared with the control. Many members within *Pseudomonadota* such as *Methylophilus*, *Methyloversatilis*, *Piscinibacter*, *Hydrogenophaga*, *Acidovorax*, *Aquabacterium*, and *Phenylobacterium* were substantially enriched after introducing PE‐derived DOM, especially black and yellow PE inputs. *Flavobacterium* belonging to *Bacteroidota* also grew rapidly after all PE‐derived DOM treatments, while black PE‐derived DOM additionally promoted *Runella* growth. *Oligoflexus* was significantly enriched after PE‐derived DOM mixture input. Besides, black and yellow PE‐derived DOM inputs were beneficial to the growth of *Cyanobium* and *Microcystis*, and the latter significantly stimulated growth of an uncultured bacterium in *Verrucomicrobiota*. Although the differences between PE‐derived DOM treatments and the control were not statistically significant, *Acidoferrum*, an oligotrophic member of family *Acidiferrobacteraceae*, exhibited lower relative abundance across PE‐derived DOM treatments, except in red PE‐derived DOM group.

**Figure 3:**
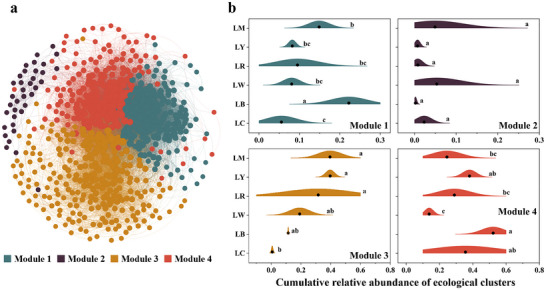
Changes in bacterial ecological associations under PE‐derived DOM treatments. a) Bacterial network based on all samples with nodes colored according to each of the four ecological clusters (module 1–4). b) Cumulative relative abundance of four ecological clusters within the constructed microbial network across different treatments. Different letters denoted significant differences in values among treatments (*p* < 0.05; One‐way analysis of variance (ANOVA), LSD post hoc test). LC represented as the natural lake water; LB, LW, LR, and LY, respectively, expressed as the lake water added the leached DOM from black, white, red, and yellow PE. LM was set as a combined treatment, where four kinds of the collected PE‐derived DOM were converged in equal volume and acted on the same bacterial community together.

Furthermore, we found there were four ecological clusters (module 1–4) strongly co‐occurring with each other based on bacterial network constructed by using all samples (**Figure** **3**a). Each ecological cluster comprised of different taxonomic profiles, mainly dominated by *Pseudomonadota*, *Bacteroidota*, *Actinobacteriota*, and *Cyanobacteriota* (Figure S10, Supporting Information). Modules 1 and 4 contained a certain proportion of negative associations, indicating potential competition or niche partitioning among taxa.^[^ ^33^ ^]^ Conversely, taxa within module 2 and 3 showed a predominance of positive associations, suggesting potential cooperation or niche sharing,^[^ ^34^ ^]^ and providing a list of potential “winner” taxa. Nevertheless, different PE‐derived DOM inputs could lead to apparent changes in the abundance patterns of interphylum bacterial associations of ecological clusters (Figure 3b). For example, adding black PE‐derived DOM strongly increased the relative abundance of taxa cluster in module 1 (including PE‐derived DOM mixture treatment), and somewhat fostered the growth of taxa in module 3 and module 4. Higher relative abundance of bacterial taxa of module 3 was present under the addition of the leached DOM from yellow or red PE, along with PE‐derived DOM mixture. Taxa involved in module 2 also exhibited a slightly enhanced metabolic efficiency when introducing white PE‐derived DOM. These results identified potential taxa subsets that are closely related to the DOM released from different colored PE debris.

### 2.3 Black PE Plastic Pollution Enhances *OXA*‐Like β‐Lactamase Genes Dissemination

We assessed the variations of ARG profiles under different PE‐derived DOM inputs by metagenomic sequencing, including ARG subtypes (categorized according to gene names), ARG types (categorized according to drug classes of antibiotics), and ARG abundance. From the control and PE‐derived DOM treatments, we recognized a total of 1341 ARG subtypes, which conferred resistance to 35 ARG types in detail, based on the Comprehensive Antibiotic Research Database (CARD). Adonis analyses confirmed a striking ARG composition (both ARG subtypes and types) discrepancy between varied treatments (*R* ^2^ = 0.953 and 0.967, *p* = 0.001, Figure S11, Supporting Information).

**Figure 4:**
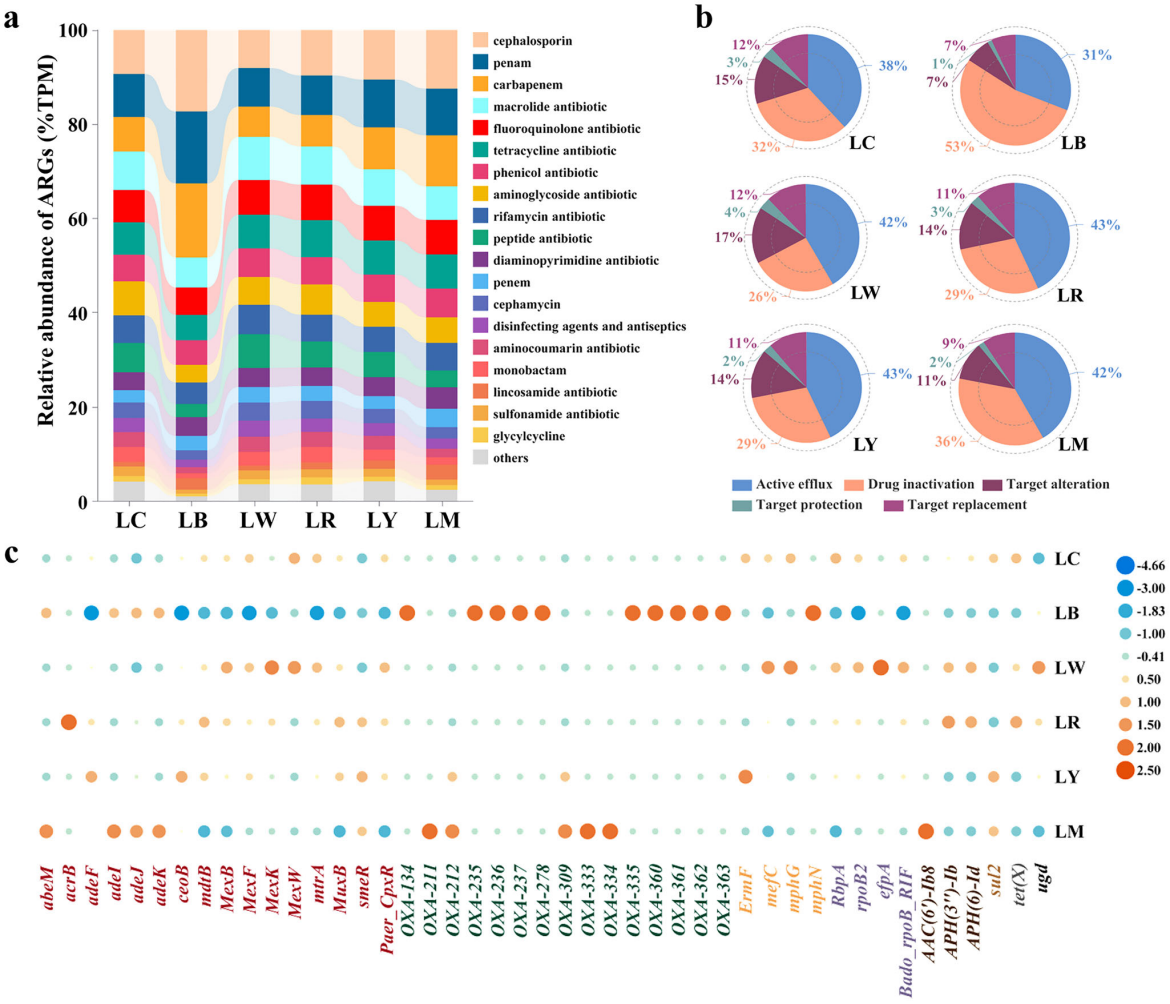
Synchronous variations of ARG profiles in bacterial communit. a) Composition profile of ARG types among treatments. b) Relative abundances (TPM) of ARGs categorized by resistance mechanisms in each microcosm, presented as means (*n* = 2). c) The heatmap showing the dynamical changes of the top 20 ARGs in each microcosm, with the values indicating the relative abundance (TPM) by log 2 transformed. Abundance values were reported as the mean (*n* = 2). ARGs with the same resistance were marked with the same color. ARGs marked red, green, yellow, purple, and brown were annotated as conferring resistance to multidrug, β‐lactamase, MLS, rifamycin, and aminoglycoside, respectively. Resistance genes *sul2*, *tet(X)*, and *ugd* were severally annotated as conferring resistance to sulfonamides, tetracycline and peptide. LC represented as the natural lake water; LB, LW, LR, and LY, respectively, expressed as the lake water added the leached DOM from black, white, red, and yellow PE. LM was set as a combined treatment, where four kinds of the collected PE‐derived DOM were converged in equal volume and acted on the same bacterial community together.

We found β‐lactamase resistance genes showed a strong association with black PE‐derived DOM input, as evidenced by a higher proportion of cephalosporin, penam, and carbapenem in its polluted microcosm (**Figure** **4**a). Also, antibiotic inactivation further replaced antibiotic efflux as the most abundant resistance mechanism (Figure 4b). By contrast, the microcosms contaminated by white, red, and yellow PE, with similar load of ARG types, preferred to activate antibiotic efflux mechanism to develop antibiotic resistance. These results could be expected as each microcosm harbored its predominant ARG subtypes. We listed the top 20 ARG subtypes with the highest relative abundance within each PE‐derived DOM treatment. Unexpectedly, we found that the β‐lactamase resistance genes dominated by *OXA*‐like gene family, exhibited absolute dominance in the microcosm enriched with black PE‐derived DOM (Figure 4c). This was a prominent observation since nearly half of dominant ARGs conferred macrolide, rifamycin, and aminoglycoside resistance among the microcosms enriched with the leached DOM from white, red, or yellow PE, as along with the control. Multidrug resistance genes were on average observed but differed by treatments. It was also worth mentioned that the combined treatment appeared to act independently and diversely. For example, some *OXA*‐like genes became dominant or peptide_*ugd* detected in single PE‐derived DOM treatment was no represented. In general, most ARGs that were identified in the treatments of white, red or yellow PE‐derived DOM, overlapped with those found in the background control, with abundance variation correlating with color‐dependent DOM heterogeneity. Black PE‐derived DOM input significantly altered the abundance and distribution of *OXA*‐like β‐lactamase genes within bacterial community.

### 2.4 Covariation between Responsive Taxa and ARGs Reflects Potential Host‐Selective Enrichment

On the basis of PCoA analysis of both abundances of OTU communities and bacterial ARGs, Procrustes test revealed a significant synchrony or coupling between the bacterial community and ARG profile in different treatments (M^2^ = 0.468, *p* < 0.01, Figure S12, Supporting Information). To further identify the responsive bacterial taxa implicated in the increase abundance of ARGs within different treatments, we constructed a positive co‐occurrence network between OTU communities and ARG subtypes, while samples from all PE‐derived treatments and the control were used (Figure S13a, Supporting Information). Thereinto, *Pseudomonadota*, *Bacteroidota*, *Actinobacteriota*, *Cyanobacteriota*, and *Firmicutes_A* were predicted as the primary potential ARG hosts since OTUs within these phyla showed high connectivity with most ARGs (Figure S13b, Supporting Information). Accordingly, *Pseudomonadota*, *Bacteroidota*, *Actinobacteriota*, *Cyanobacteriota*, and *Firmicutes_A* may play key roles in aquatic resistance dissemination under the inputs of diverse PE‐derived DOM. Based on the modularity class, with 94.4% of total vertices in entire network occupied by the top eight modules, which would explain most of the information of the network. We then summed the abundance of both bacterial OTUs and ARGs within these modules (Figure S14, Supporting Information). Their similar abundance trends could suggest that ARG selection driven by PE‐derived DOM input was taxon‐specific.^[^ ^35^, ^36^ ^]^

**Figure 5:**
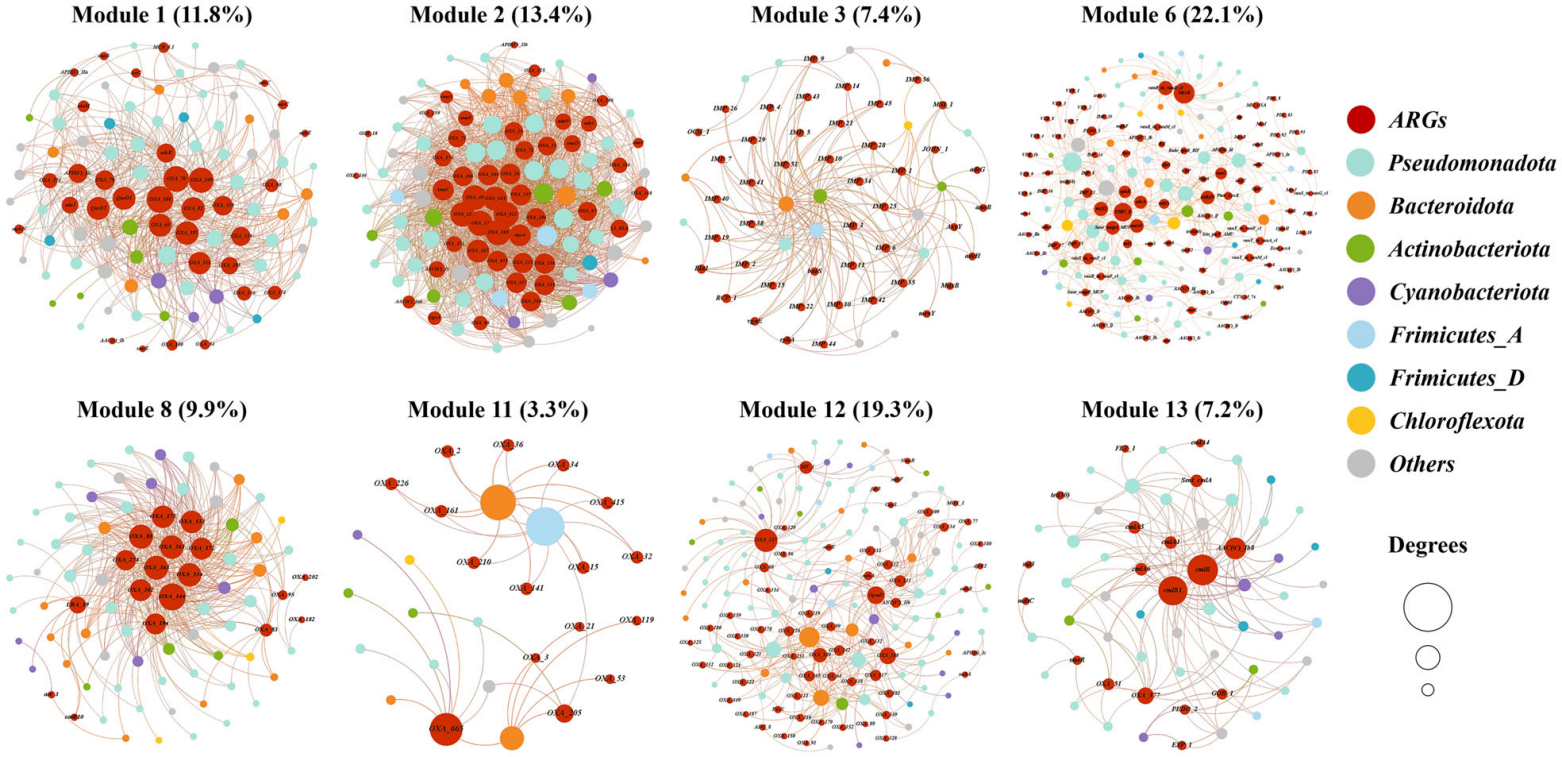
Eight dominant modules in the positive co‐occurrence network among ARG subtypes and bacterial OTUs. Node colors are assigned based on ARG subtypes and bacterial taxa at the phylum level, and node sizes correspond to degree (number of connections). The numbers in parentheses represent the proportion of each module in the entire network.

Module units further divided the specific co‐occurrence relationship between detailed ARG subtypes and possible hosts (**Figure** **5**). *OXA*‐like β‐lactamase genes seemed to be more independent of PE‐derived DOM treated ecological clusters, particularly those benefit from black PE‐derived DOM. This could be evident by the more of the ARG abundance driving the response to black PE‐derived DOM input in module 1, 2, 8, and 11. Thereinto, module 1, 2, and 8 were tightly nested into a larger module, showing a strong intermodular association. Another partial *OXA*‐like genes cluster was shared by other four PE‐derived DOM treated samples in module 12, but more abundant under PE‐derived DOM mixture input. Given these, black PE‐derived DOM had a broader influence on the potential host clusters of *OXA*‐like β‐lactamase genes. Furthermore, although with the similar cluster abundance, module 13 was distinguished from module 12 through recruiting more ARGs associated with the antibiotic efflux mechanism. Taxa within module 13 may utilize the available DOM leached from white, red, and yellow PE to support their growth and develop the resistance genes related to antibiotic efflux. Module 3 more reflected a possible low frequency stimulation of DOM leached from red, yellow, and white PE on some sensitive species, with the consequence of higher relative abundance of some *IMP* genes (conferring β‐lactamase resistance). By contrast, module 6 placed emphasis on the background resistotype covering multiple ARG families affiliated with β‐lactamase (*THIN‐B*, *PDC*‐, *VEB*‐, *IMP*‐families), aminoglycoside (*AAC*‐, *APH*‐families), and glycopeptide (*van*‐families). Overall, when diverse PE‐derived DOM were introduced into the aquatic systems, bacterial differential responses resulted in unique community dynamics, thereby altering ARG profiles and ultimately enhancing resistance transmission.

### 2.5 Labile PE‐Derived DOM Inputs Are Linked to Elevated Metabolic Exchange and Synergistic Resource Reallocation

Abundance‐based bacterial community succession explained the changes in ARG profiles under different PE‐derived DOM inputs, which basically depends on the metabolic utilization behavior of bacteria to the available resource in the environment.^[^ ^37^ ^]^ We first understood the overall composition of DOM metabolites when the microcosm experiments were finished. PARAFAC analysis identified five fluorescent components for EEM dataset of lake water DOM samples (Figures S15 and S16, Table S4, Supporting Information), and changes in fluorescence intensity of each component were at a smaller margin before and after the incubation (Figure S17, Supporting Information). However, the observed richness of DOM metabolites obviously decreased in the microcosms enriched with black PE‐derived DOM and PE‐derived DOM mixture (Figure S18, Supporting Information). This indicated many DOM molecules may be generated through biological processes in conjunction with hydrolysis.^[^ ^38^ ^]^ For per treatment, most identified DOM molecules displayed lignin‐like compositions and saturated compounds, and analyzing the elemental composition of these formulas, CHO and CHON subcategories were the most abundant (Figure S19, Supporting Information). Although the composition of DOM metabolites across treatments remained comparable, their relative abundances exhibited significant temporal variations between day 0 and 7 (Figure S20, Supporting Information), reflecting the DOM‐dependent metabolic adaptations of bacteria.

**Figure 6:**
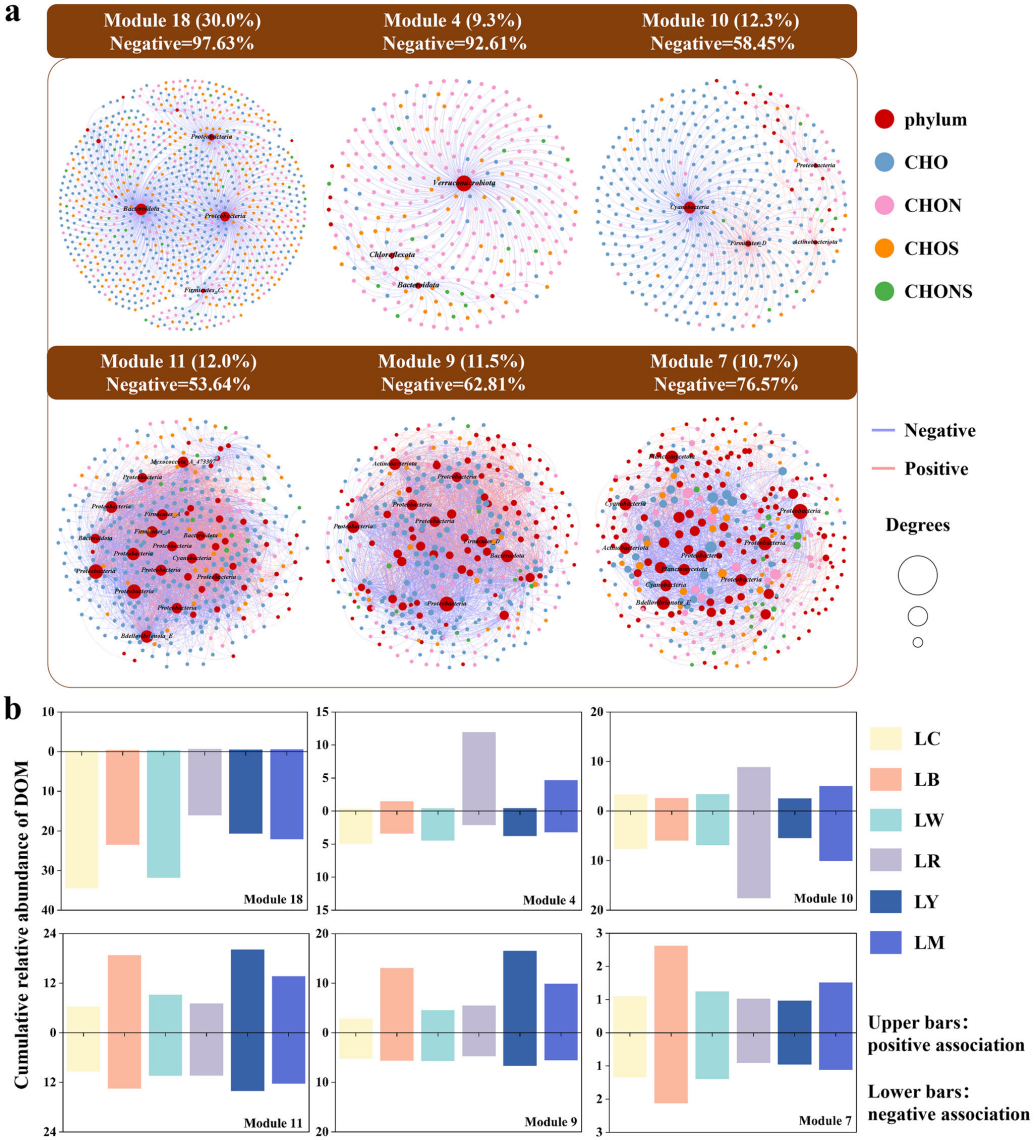
Bacterial interactions with DOM molecules. a) The top six main ecological clusters with nodes colored according to bacterial phyla level and DOM elemental composition (CHO, CHON, CHOS, and CHONS). The numbers in parentheses represent the proportion of each module in the entire network. Node sizes correspond to degree (number of connections). b) The cumulative relative abundance of DOM compounds in different ecological clusters under different treatments. The upper bars represent the cumulative relative abundance of DOM molecules that were positively correlated with bacterial OTUs, whereas the lower bars represent those that were negatively correlated with the corresponding OTUs. LC represented as the natural lake water; LB, LW, LR, and LY, respectively, expressed as the lake water added the leached DOM from black, white, red, and yellow PE. LM was set as a combined treatment, where four kinds of the collected PE‐derived DOM were converged in equal volume and acted on the same bacterial community together.

Likewise, we considered all samples and then correlated the relative peak intensity of DOM molecules and the relative abundance of bacterial OTUs. The network exhibited reliable modularity and the top six of the twenty‐five total modules explained 85.7% of all vertices (Figure S21, Supporting Information). Among these modules, module 18 and 4 were dominated by negative associations, while module 11, 10, 9, and 7 increased more positive correlations (**Figure** **6**a). Positive and negative interactions in the network reflect covariation between bacterial OUTs and DOM metabolites, serving as hypothesis‐generating indicators of DOM accumulation or consumption.^[^ ^31^ ^]^ Each module displayed distinct DOM molecular composition and bacterial taxa (Figure S22, Supporting Information), representing a unique functional unit involved in putative DOM transformation.^[^ ^39^, ^40^ ^]^ According to connectivity within and among modules (Figure S23, Supporting Information), bacterial OTUs provided all the network hubs and the majority of module hubs in the network, which were assigned as *Pseudomonadota* accounted for ≈43.1%, followed by *Bacteroidota* (13.8%) and *Actinobacteriota* (8.6%). Most connectors belonged to DOM compounds were dominated by CHO formulas (64.7%), which may be key intermediate substrates or products from bacterial metabolic activities. Statistically, ≈83.2% of these DOM connectors were statistically distributed in module 11, 9, and 7. The cumulative abundance changes between different treatments were further compared to explore the potential metabolic strategy of module taxa to distinct PE‐derived DOM (Figure 6b; Figure S24, Supporting Information).

Module 18 contained the highest numbers of DOM molecules mainly classed as lignin‐like compounds (with CHO elemental composition). They showed strong negative correlations with two members belonging to the environmental ubiquitous dominant phyla, *Pseudomonadota* and *Bacteroidota*, exhibiting a higher abundance in the control and white PE‐derived DOM treatment. As showed in module 10, many DOM molecules showed a strong negative correlation with the specific species belonging to *Cyanobacteriota*. Meanwhile, most of the remaining positive interactions mainly occurred in *Firmicutes_D* and *Actinobacteriota*, which were more proactive in respond the red PE‐derived DOM. *Verrucomicrobiota* dominated module 4, with several other taxa. Except for the observation of their almost negative correlations with specific DOM molecules (most fell into CHON class). We additionally noticed that *Verrucomicrobiota* was positively correlated with some molecules with CHOS elemental composition. These organic S compounds enriched in the microcosms containing the red PE‐derived DOM. The most complicated interactions were present in module 7, 9, and 11, involving abundant bacterial taxa and keystone module members. The DOM components released by black and yellow PE, would have a higher affinity by these three taxa clusters, acting as an important substrate for bacteria metabolism. As a result, we observed a higher cumulative abundance of positively correlated DOM in module 7, 9, and 11, suggesting a potential increase of bacterial metabolic efficiency.

## 3 Discussion

Plastic pollution is among the pressing environmental issues of our time.^[^ ^10^ ^]^ Against this background, plastic‐derived DOM has become an increasingly significant contributor to the natural organic carbon pool in aquatic ecosystems.^[^ ^23^ ^]^ However, their actual inputs can be strongly but variably influenced by plastic colors, as evidenced by our results, supporting a few reports of color as a key role in plastic photoaging.^[^ ^25^, ^41^, ^42^ ^]^ Red PE released more DOC and greater amounts of humic‐like components than other colored PE. This is in line with the expectation. Color with longer wavelength enables more efficient capture of high‐energy ultraviolet photons to accelerate plastic photodegradation,^[^ ^42^, ^43^ ^]^ as evidenced by our SEM and FTIR results. Accordingly, white, black, and yellow would likely help plastics undergo slower photoaging, thereby enhancing environmental persistence of PE plastic bags and sustainably releasing organic matter over a long period of time. Furthermore, given that yellow, black, and white PE debris leached more labile DOM, these plastics would likely supply greater bioavailable carbon to support bacterial growth in surrounding waters. Consequently, as one of the most common characteristics of plastic wastes in aquatic environments, plastic color also may indicate the environmental fate and potential ecological implications of plastic‐derived DOM.

Our maintainable microcosm experiments reproduced the previous result that PE‐derived DOM can stimulate bacterial growth.^[^ ^14^, ^15^, ^20^ ^]^ Lake bacterial communities rapidly altered their metabolic strategies and taxonomic composition in response to short‐term nutrient enrichments (i.e., introduction of PE‐derived DOM). According to the characterization of bacterial community composition, phylum *Pseudomonadota* benefited the most from the available plastic‐derived DOM, with an increased abundance. This is common and reasonable since many members of *Pseudomonadota* are copiotrophic, following the *r*‐strategist (owning a fast growth rate and a rapid response to available carbon and nutrient inputs).^[^ ^44^, ^45^ ^]^ Therefore, these copiotrophs are strongly selected in most cases after nutrient addition, as also supported by the enrichment of some genera led by *Methylophilus* and *Piscinibacte* that were observed in our study. More broadly, when plastic‐derived DOM exposes to aquatic environments, their bioavailable parts could be rapidly eliminated by fast‐growing copiotrophic taxa in the short term. This would contribute to help direct biological remediation strategies to reduce chemical pollution from plastics in the environment, as suggested in previous study.^[^ ^15^ ^]^ At the same time, bacterial diversity analyses revealed that the sustained enrichment of copiotrophic taxa, primarily represented by *Methylophilus* and *Piscinibacter* (*Pseudomonadota*), together with contributions from *Cyanobium* and *Microcystis (Cyanobacteriota)*, order *Methylacidiphilales* (*Verrucomicrobiota*), and *Oligoflexus* (*Bdellovibrionota_E*), markedly altered the initial community balance. Different changes in community structure reflected the metabolic flexibility of the same species pool to available resources of different PE‐derived DOM. Black and yellow PE‐derived DOM have a stronger influence on community composition by enriching their preferred taxa.

We now advance beyond this work by also showing that the release of PE‐derived DOM into the environment has the potential to influence the spread of antibiotic resistance genes.^[^ ^46^ ^]^ Of further concern, *OXA*‐like genes conferring β‐lactamase resistance became more abundant and diversified in the microcosm enriched with DOM released by black PE. By contrast, only a slight increase in the abundance of certain genes associated with antibiotic efflux mechanism were observed in the microcosms enriched with the other three PE‐derived DOM. Most ARGs in these treatments overlapped with those detected in the background control. Accordingly, our results underscore the concerns regarding the potential dissemination of β‐lactamase resistance in aquatic environments polluted by black PE plastics. It should be noted that the *OXA*‐like β‐lactamase genes reported here were solely identified by database sequence similarity, which cannot establish carbapenemase activity and request for further isolate phenotyping analysis to confirm clinical relevance. On the other hand, although the impacts of other colored PE‐derived DOM on ARG profiles within bacterial communities were modest, they should also not be taken lightly considering the potential for continuous, long‐term leaching of plastic‐derived DOM. While current attention often centers on biofilm‐associated “plastisphere” risks,^[^ ^47^, ^48^, ^49^ ^]^ our findings highlight that the restructuring of heterotrophic communities driven by plastic‐derived DOM represents a parallel pathway by which plastic pollution can also amplify ARG reservoirs and their dissemination potential.

Network analysis can help characterize metabolic trade‐offs in bacterial communities in response to inputs of plastic‐derived DOM, an aspect that remains underexplored. Herein, we combined data across treatments to construct three metanetworks, each mapping cross‐treatment association patterns among studied elements.^[^ ^50^, ^51^, ^52^ ^]^ Notably, as a tool for interaction prediction, such network inference is hypothesis‐generating rather than explanatory.

Bacterial co‐occurrence network recognized four ecological clusters of important taxa, revealing similar bacterial association patterns under varying resource availability.^[^ ^33^ ^]^ The relative abundances of these ecological clusters were selectively sensitive to the different PE‐derived DOM inputs. Black PE‐derived DOM had broader putative beneficiary clusters than those from the other three colored PE. It is thus supposed that the intertaxon potential interactions are more vulnerable to the black PE plastic pollution. Moreover, ecological positive associations were prevalent among bacterial taxa, implying that naturally occurring bacteria populations may tend to coexist rather than compete when responding the inputs of DOM derived from colored plastics.^[^ ^53^ ^]^ Although the exact specific mechanisms underlying these associations are unknown with correlation‐based network analyses, the intensity and direction of bacterial taxa potential interactions may be undergoing changes under the color‐driven plastic pollution.

More importantly, differential bacterial metabolic adaptation to distinct PE‐derived DOM inputs may have modulated ARG dynamics by altering nutrient utilization patterns and interspecific interactions within the community. Although no single mechanism could be conclusively assigned due to the limitation of short‐read datasets, the concurrent increase of β‐lactamase genes (especially *OXA*‐like genes) and their putative hosts (e.g., *Methylophilus* and *Piscinibacter*), is consistent with selective enrichment as one plausible explanation. Previous studies have shown that fast‐growing taxa often act as ARG carriers under nutrient‐enriched conditions, given their nutrient‐driven advantages and frequent association with mobile genetic elements, thereby accelerating dissemination of antibiotic resistance.^[^ ^54^ ^]^ As a result, some abundant copiotrophic genera within *Pseudomonadota*, *Bacteroidota*, and *Actinobacteriot*a are frequently implicated as main ARG hosts.^[^ ^55^, ^56^ ^]^ Consistent with these findings, our results highlight the opportunistic members within *Pseudomonadota* that showed strong positive correlations between their relative abundance and the occurrence of *OXA*‐like β‐lactamase genes. Although co‐occurrence alone cannot confirm selective enrichment, coselection, or horizontal gene transfer, this hypothesis‐generating framework provides valuable insights for prioritizing key taxa and ARGs for subsequent mechanistic validation. Overall, color‐mediated variations in PE‐derived DOM composition may not directly induce host‐specific differentiation of ARGs, but it likely drives community‐level selective processes favoring copiotrophic and opportunistic bacteria. Such dominance may further intensify ecological competition for resources and potentially influence the energetic costs associated with maintaining antibiotic resistance.^[^ ^57^, ^58^ ^]^

Resource availability fundamentally constrains community dynamics. The consumption and accumulation of DOM are a result of interactions between the diverse bacterial community and DOM molecules.^[^ ^59^, ^60^ ^]^ Although the putative significant correlations between taxa and DOM molecules in bacteria–DOM network are not direct evidence of metabolic transformation, such correlation analyses at least provide initial insights for future studies into microbe–DOM coupling and bidirectional feedback dynamics. In our study, some copiotrophic members of *Pseudomonadota* and *Bacteroidota* showed broad substrate affinity, as suggested by inferred negative associations with PE‐derived DOM constituents. Accordingly, they can be the favorable eliminator when various plastic wastes release DOM into the aquatic environments. Likewise, several members of *Firmicutes_D* and *Actinobacteriota* were negatively associated with some recalcitrant DOM components, which is compatible with potential degradation or consumption. In fact, special taxa within *Actinobacteriota* (e.g., *Streptomyces*) and *Firmicutes* (e.g., *Bacillus*) have been shown to degrade recalcitrant organic matter via specialized enzymatic and metabolic pathways.^[^ ^61^ ^]^ Given the observed putative, mutually beneficial associations with heterotrophic bacteria, certain *Cyanobacteriota* taxa may act as “gleaners.”^[^ ^62^ ^]^ Moreover, we suppose their mutualistic interaction may be strengthened with increasing nutrient supply due to higher *Cyanobacteriota* abundance observed in microcosms adding PE‐derived DOM. On the other hand, selective associations between particular *Verrucomicrobiota* and S‐containing organic matter may indicate a potential contribution to sulfur cycling, offering tentative insight into how plastic pollution could influence ecosystem sulfur processes. Recently, taxa within *Verrucomicrobiota* have been shown to encode and express genes for S metabolism pathways,^[^ ^63^ ^]^ yet little else is known. More importantly, our findings highlight that bacterial metabolic exchanges are dynamic and important in the color‐driven plastic pollution, although they are ubiquitous in microbial communities.^[^ ^64^ ^]^ Diverse functional taxa gather DOM compounds of differing traits, and putative keystone taxa exhibited bidirectional (positive and negative) associations with these compounds. This suggests a metabolite‐exchange‐like pattern among bacteria, which potentially performs similar metabolic complementarity among taxa and functional complementarity among the network modules.^[^ ^65^ ^]^ Remarkably, labile PE‐derived DOM from black and yellow debris may contribute to a possible amplification of metabolic exchange complexity in interacting bacterial populations via preferential stimulation of copiotrophs, based on similar metabolite‐mediated facilitation.

**Figure 7:**
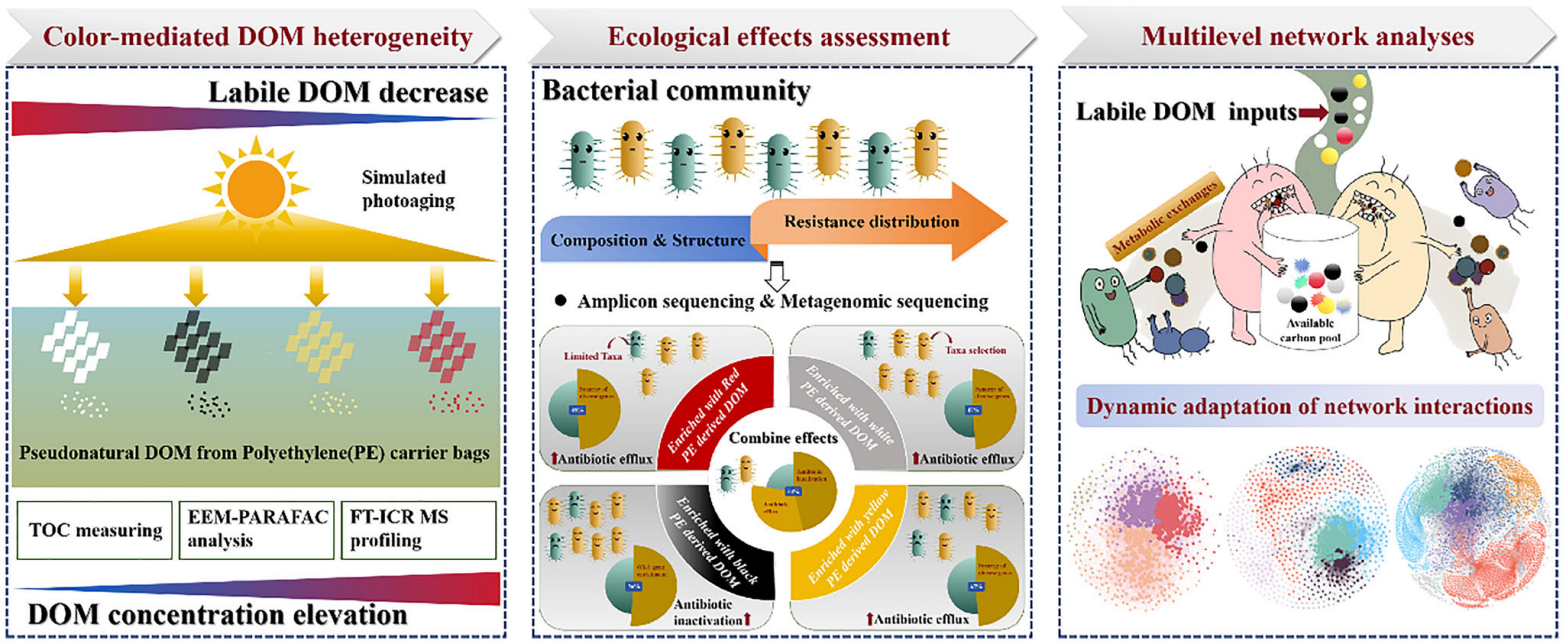
Conceptual illustration depicting color‐mediated release heterogeneity of PE‐derived DOM from colorful carrier bags during photoaging and the metabolic adaptations bacterial community taxa in response to their inputs, causing modulation of bacterial interactions and antibiotic resistance dynamics.

Taken together, our results revealed how plastic color influences the chemical composition and bioavailability of plastic organic carbons, as well as its subsequent potential implications on bacterial community and transport dynamics of antibiotic resistance (**Figure** **7**). These findings well support the previous viewpoint that it is inappropriate to extrapolate of leaching behaviors and environmental risks only from white or transparent plastics to various colored plastics. Substrate‐use strategies vary among bacterial lineages. Accordingly, the color‐dependent chemical heterogeneity of PE‐derived DOM imposed selective pressures on bacterial community by supporting taxa with compatible resource‐use traits, inducing taxonomic filtering effects. In particular, labile DOM released from black PE plastics stimulated the growth of several copiotrophic taxa within *Pseudomonadota* by improving substrate availability, further resulting in an enhanced expression of β‐lactamase resistance within bacterial community. More broadly, our study emphasizes that plastic‐derived DOM can favor fast‐growing copiotrophic taxa, and potentially tilt community toward copiotrophic profile with sustained inputs, thereby affecting the spread of antibiotic resistance. Putative significant correlation indicated that fast‐growing copiotrophs supported by labile PE‐derived DOM, potentially provide more nutrient availability for other co‐occurring taxa by metabolic exchanges, in turn displaying a potential modulation on host‐selective ARG enrichment. These outcomes highlight the importance of understanding plastic‐derived DOM in shaping community structure and ecological function from a network perspective. However, plastics are highly diverse in reality. They can consist of various polymers; include a broad array of chemical additives; come in many shapes, sizes, and colors.^[^ ^66^ ^]^ Hence, there is a mass of different factors to consider, each probably differing to some extent in their effects on plastic leaching behaviors, with the consequence of chemical diversity in plastic‐derived DOM. Our findings just help to complement current understanding of the ecological implications of discarded PE plastic bags via DOM release by highlighting the role of color diversity, while offering additional valuable insights for guiding sustainable production practices. Meanwhile, short‐term microcosm observation may limit the generalizability of our conclusions, as the findings primarily reflect early‐phase community and ARG responses. Longer‐term dynamics, such as community succession, ARG persistence, and seasonal variability, may differ in both direction and magnitude.

## 4 Conclusion

This study provides the first insights into the connection among heterogeneity of plastic‐derived DOM, shifts in community composition and selection for antibiotic resistance, together with characterizing the putative ecological relationships that may couple these changes. Compared with the other three colors (i.e., white, red, and yellow), black PE plastic pollution is more likely to destabilize the microecological balance (especially stimulating copiotrophic taxa within *Pseudomonadota* growth) and increase the transmission risk of *OXA*‐like β‐lactamase genes. It thus should be paid more attention to in future pollution managements. Besides, network analyses reveal a putative ecological interaction pattern in PE‐polluted aquatic environments. Labile PE‐derived DOM inputs (especially from black and yellow PE) are associated with enhanced metabolic exchange and a synergistic reallocation of available resources, thereby potentially modulating bacterial interactions and ARG–host linkages. Overall, our work extends the traditional focus on “plastic size” and highlights color as a multidimensional driver of plastic pollution. Higher taxonomic and genomic resolution will be required in future works to better resolve strategy‐specific responses, given numerous unclassified genera and metabolic diversity within phyla.

## 5 Experimental Section

### Collection of Four PE‐Derived DOM

PE carrier bags in four colors (black, white, yellow, and red) were purchased from a local farmers market and uniformly cut into 1 cm × 1 cm square debris with thickness ≈0.05 mm. Every 5 g PE debris was placed in precombusted glass flasks with 300 mL distilled water and irradiated for 14 days, as well as constant agitation to build environmental fluctuation. During this period, it was suggested to shake the glass flasks regularly to make sure the PE debris were irradiated evenly. Irradiation environment was equipped with six UVA‐340 lamps (280–400 nm wavelength) as light sources to imitate natural ultraviolet exposure. Irradiation experiments were performed in triplicate. Three separate precombusted flasks of 300 mL of distilled water, without adding any plastic debris, were synchronously established as background control under the same condition, in order to confirm no plastic contamination of leaching environment. In the end of irradiation, severally, each plastic leachate solution was filtered through prerinsed 0.45 µm glass fiber filter to obtain ultimation PE‐derived DOM for the next characterization and microcosm experiments.

### Microcosm Experiment Overview

In September 2023, lake samples for microcosm construction were collected in Foshan University, southern China (23.02 N, 113.06 E). The lake water was immediately filtered on site. The 0.22 µm sterile filter membranes intercepting microorganisms and the 0.45 µm filtrate were then sent to the laboratory to determine the bacterial community composition and general characteristics of the sampled lake.

After filtering by 200 µm sieve to remove larger plankton and particles, the collected lake water was randomly injected into six glass tanks precleaned with 0.1% K_2_MnO_4_ and distilled water, with average water volume of 8 L per tank. After placing for 3 h to stabilize the microbial community, four tanks severally received 2 L of DOM filtration generated by each color PE debris, and a tank was injected an additional 2 L of sterile distilled water as the control. Meanwhile, a combined treatment containing four kinds of PE‐derived DOM filtration was also designed to better reflect the reality of coexisting of colorful plastics. Four kinds of collected PE‐derived DOM filtration were pooled together at equal volumes (500 mL) to prepare a final mixed DOM filtration, which was subsequently added into the sixth tank labeled as combined treatment. Each microcosm was covered with breathable sealing film for air exchange and incubated for 7 days at ambient temperature, a duration shown to resolve early, measurable physiological and compositional changes in bacterial communities exposed to plastic‐derived DOM inputs.^[^ ^14^, ^67^, ^68^ ^]^ The adopted volume of PE‐derived DOM samples expressed that bacterial communities were exposed to 3.33 g plastic/L, which was in line with the current recorded actual environmental concentration of plastics.^[^ ^69^ ^]^

Enriching microorganisms was performed by using sterile filter membranes (45 mm, 0.22 µm pore size). For each of the six tanks, lake water samples were successively filtered through 3 µm and 0.22 µm sterile filter membranes at the end of incubation. Ten obtained filter membranes for each tank were then placed into 50 mL sterile tubes and frozen at −80 °C, used for further 16S rRNA gene metabarcoding sequencing and metagenomic sequencing. At the same time, an additional 1.5 L of lake water taken from each tank was filtered through 0.45 µm glass fiber filter membrane, and six groups of lake filtrate samples were acquired for chemical characterization. For each group of lake filtrate samples, 3 × 10 mL samples were for DOM concentration measurements, 3 × 50 mL samples for DOM fluorescence analyses, 3 × 50 mL samples for DOM FT‐ICR MS tests; another set of 3 × 150 mL samples were for general physicochemical properties analyses, involving DON, phosphate (PO_4_^3−^) and inorganic nitrogen (NH_4_^+^, NO_3_^−^, NO_2_^−^).

### DOM Characterization

DOM was characterized by DOC quantification, optical EEM‐PARAFAC analysis, and ultrahigh‐resolution FT‐ICR MS for molecular composition.

i) DOC measurement and DOM optical characterization

Concentration of total DOM in the filtration was expressed as the DOC concentration, which was determined using multi N/C 2100 TOC analyzer (JENA, Germany), as described in Section S1 of the Supporting Information. 3D excitation emission matrix (3D‐EEM) of DOM samples was recorded with a fluorescence spectrometer (HORIBA Aqualog, version 3.6, Japan). PARAFAC analysis of the 3D‐EEM spectral data was conducted using the DOMFluor toolbox in MATLAB R2022b to characterize fluorescent signatures of DOM. Distinct PARAFAC models identified four components for PE‐derived DOM and five components for lake DOM (Figures S3 and S16, Supporting Information), with model performance confirmed by standard validation procedures. Details of the spectral scanning setup and interpretation about model results are provided in Section S2 of the Supporting Information.

ii) DOM FT‐ICR MS analysis and molecular identification

DOM samples underwent solid‐phase extraction and were subsequently analyzed by ultrahigh‐resolution FT‐ICR MS (7 T Bruker XR) for molecular identification.^[^ ^15^ ^]^ FT‐ICR MS was equipped with an electrospray ionization source, with negative detection mode. Raw MS files were processed in Bruker DataAnalysis (version 5.0) for generation of m/z peak lists with signal‐to‐noise ratio ≥ 6, followed by internal calibration. Detailed information regarding the SPE processes and FT‐ICR MS test are provided in Section S3 of the Supporting Information.

Molecular formulas of all mass peaks were assigned in the FTMSAnalysis platform,^[^ ^70^ ^]^ and summarized by four elemental combinations (CHO, CHON, CHOS, and CHONS). In van Krevelen diagram, these assigned molecules were additionally categorized into seven compound classes based on O/C and H/C element ratios, including lignins, saturated compounds, aminosugars, carbohydrates, tannins, unsaturated hydrocarbons, and condensed aromatic structures. Some molecular traits (M/Z, O/C, H/C, DBE, NOSC, and AI_Mod_) were also calculated and expressed as the intensity‐weighted means (Table S2, Supporting Information). H/C ratio ≥ 1.5 was employed as an operational indicator for characterizing the labile constituents within DOM compounds.^[^ ^71^ ^]^ The relative abundance of molecular formula was calculated by normalizing signal intensities of assigned peaks to the sum of all intensities within each sample, and reported as triplicate means representing the ultimate relative abundance of identified DOM compounds per treatment. A detailed description for the processing of DOM‐related FT‐ICR MS data is available in Section S4 of the Supporting Information.

### 16S rRNA Gene Metabarcoding Sequencing and Metagenomic Sequencing

Bacterial community composition and ARG profiles were determined by 16S rRNA gene metabarcoding sequencing and metagenomic sequencing, respectively.

i) DNA extraction

Bacterial genomic DNA was extracted from filter membranes. After assessments of DNA concentration and purity, the resulting DNA sample for each treatment was divided into two parts for the following 16S rRNA gene metabarcoding sequencing and metagenome sequencing. More details concerning DNA extraction and DNA library construction are given in Section S5 of the Supporting Information.

ii) 16S rRNA gene metabarcoding sequencing

Bacterial community composition was characterized by 16S rRNA gene metabarcoding (V3–V4) on an Illumina NovaSeq platform using paired‐end 2 × 250 bp strategy. Following the cleaning and quality control of raw reads in Quantitative Insights Into Microbial Ecology‐2 (QIIME 2, http://qiime2.org/), sequences with 97% similarity were clustered into OTUs and taxonomically annotated against the Greengenes2 Database to generate the OTU table.^[^ ^72^ ^]^ The relative abundance of OTUs was calculated by the normalization of read counts of OTUs to the sum of all reads within each sample. Bacterial diversity was further evaluated on the basis of OTU taxonomic ranks and abundance data. α‐Diversity was calculated using the Chao1 species richness (i.e., the total number of OTUs in each sample), Shannon index (higher Shannon index referring to higher diversity), and Simpson index (with a high sensitivity to dominant species).^[^ ^49^ ^]^ β‐Diversity was assessed by Bray–Curtis, and group differences were examined by PERMANOVA. Each treatment comprised three biological replicates, for a total of 18 metabarcoding sequencing samples.

iii) Metagenomic sequencing

Shotgun metagenomes from total 12 samples were sequenced on an Illumina NovaSeq (PE 150) and used for ARG profiling. Raw metagenomic data of 9.3 × 10^9^ base (3.1 × 10^7^ reads) on average were obtained (*n* = 12). They were quality controlled using the KneadData pipeline to generate the clean dataset (an average reads of 2.9 × 10^7^ per sample, Q20 = 98.7% ± 0.2% and Q30 = 95.7% ± 0.4%) for subsequent ARG annotation.^[^ ^73^ ^]^ ARGs were annotated against the Comprehensive Antibiotic Resistance Database (CARD) using DIAMOND with default settings.^[^ ^74^ ^]^ In this study, ARG annotations were reported at two levels, namely, subtypes (individual gene names) and types (antibiotic drug classes). The relative abundance of ARG per sample was calculated as TPM (length‐normalized counts per million reads). ARG profile similarities among samples were computed using Bray–Curtis distances. More detailed sequencing procedure and raw reads cleaning process are found in Sections S6 and S7 of the Supporting Information.

### Co‐Occurrence Networks

Network analysis has developed as a powerful approach in microbial ecology by providing a framework to capture the complicated ecological relationships in bacterial communities.^[^ ^75^ ^]^ The study constructed three types of meta co‐occurrence networks. For each network, following previous studies, all samples covering all PE‐derived DOM treatments and the normal control were considered, to explore the interconnection patterns between variables across processing conditions.^[^ ^50^, ^51^, ^52^, ^53^ ^]^ Spearman's rank correlation coefficient between variables based on normalized relative abundance was computed by using the hmisc package in R version 4.3.1, and the significant correlations (|*r*| > 0.7 with adjusted *p* < 0.01) were visualized using the interactive platform Gephi version 0.10.1 with the Fruchterman–Reingold layout. The *p* values were adjusted with the Benjamini–Hochberg method to reduce the frequency of false positive results.

i) Bacterial interaction network

Bacterial co‐occurrence network was defined by the Spearman rank correlation between all pairs of the bacterial OTUs, in which nodes represent bacterial OTUs (label displaying phylum level) and edges reflect the significant co‐occurrence between nodes. The focus was on the dominant OTUs that accounted for over 80% of the relative abundance in total community. Such filtering was beneficial to reducing potential spurious correlation from the rare OTUs. Modularity and ecological clusters were determined using Gephi platform (Figure S10, Supporting Information), and then the relative abundance of all divided ecological clusters was estimated.

ii) Co‐occurrence pattern among ARGs and bacteria

Network analysis of bacteria and ARGs was utilized to inferring the potential hosts of resistance genes.^[^ ^36^ ^]^ To this end, the focus was on the positive correlations between ARGs and bacteria. After visualization on the Gephi platform, the positive network was subsequently extracted and modularity was then defined for each node (representing an ARG or a bacterial OTU). This entire positive network was separated into 24 cohesive smaller modules as depicted in Figure S13 of the Supporting Information.

iii) Bacteria‐DOM correlation network

Bacteria‐DOM network was constructed based on the calculated Spearman's rank correlations between the relative abundances of bacterial OTUs and relative peak intensity of DOM molecules. To ensure statistical robustness, only bacterial OTUs and DOM molecules that simultaneously occurred in at least ten samples were included in the analyses. For bacteria–DOM network, bacterial OTUs and DOM molecules with high correlations were set as nodes, and direct edges were made from source (bacterial OTUs) to target (DOM molecules) based on correlation. Twenty‐five densely connected communities (i.e., network modules) in the network were synchronously determined as shown in Figure S21 of the Supporting Information. The number and categories of bacterial OTUs and DOM molecules were counted within modules of primary concerns (i.e., the first six modules). Besides, the roles of the network nodes were assigned according to the calculation of within‐module (Zi) and among‐module connectivity (Pi).^[^ ^76^ ^]^ Four categories were defined for each node: 1) peripheral nodes (Zi ≤ 2.5, Pi ≤ 0.62), 2) connectors (Zi ≤ 2.5, Pi > 0.62), 3) module hubs (Zi > 2.5, Pi ≤ 0.62), and 4) network hubs (Zi > 2.5, Pi > 0.62). Network hubs, module hubs, and connectors are generally considered to be potential ecosystem keystone module members.^[^ ^77^ ^]^

### Statistical Analysis

Data were presented as mean ± standard deviation for three replicates (*n* = 3), except for metagenomic sequencing, for which *n* = 2 per treatment. Data analyses were performed using SPSS version 24.0. Prior to statistical testing, data were examined for normality (Shapiro–Wilk test) and homogeneity of variance (Levene's test). When necessary, data were log‐transformed or normalized to meet the assumptions of parametric analyses. The unpaired Student's *t*‐test was used to analyze the pairwise difference. Differences among the multiple groups were evaluated using One‐way ANOVA followed by LSD post hoc test for paired comparisons. Kruskal–Wallis nonparametric analysis of variance was utilized if the data were not normally distributed. *P* < 0.05 was considered statistically significant. PCoA analysis, PERMANOVA analysis (based on Adonis function), and Procrustes analysis were implemented in R version 4.3.1 with packages “vegan” and “ggplot2.” The dissimilarities (using Jaccard distance) in community composition among treatments (β‐diversity) were partitioned into replacement (labeled as Repl) and richness difference (labeled as Richdiff) components using R package “adespatial.”^[^ ^78^ ^]^ Network analysis was performed with “vegan,” “igraph,” and “hmisc” packages in R version 4.3.1 and network graphs were visualized using Gephi version v 0.10.1. Heatmaps and Venn diagrams were constructed using TBtools version 2.154. All bar charts, scatter diagrams, box plots, and violin plots were plotted using Origin version 2024.

## Conflict of Interest

The authors declare no conflict of interest.

## Author Contributions

All authors contributed intellectual input and assistance to this study. The original concepts were conceived by S.F., S.L., and G.O. Sampling collection, water chemical and microbial characterization were carried out by S.F., J.H., and Y.H. Data collection, analyses, and integration were done by S.F., with the assistance provided by C.C. and Q.R. Technical help and guidance were provided by P.G. The manuscript was prepared by S.F., with substantial input from S.L. and G.O. All authors read and approved the manuscript.

## Supporting information



Supporting Information

## Data Availability

The data that support the findings of this study are available in the supplementary material of this article.
