# Supplementary material for: Colored Plastic Pollution Reshapes Aquatic Microbial Community and Resistance Profile via Divergent Pseudonatural Dissolved Organic Matter
Source: Adv Sci (Weinh). 2025 Oct 30;13(3):e14529. doi: 10.1002/advs.202514529 (PMC12806204; doi:10.1002/advs.202514529)
Supplement: Supplementary file 1 — Supporting Information [file ADVS-13-e14529-s001.docx]

***Supplementary information for***

**Colored-Plastic Pollution Reshapes Aquatic Microbial Community and Resistance Profile via Divergent Pseudonatural Dissolved Organic Matter**

Shuting Fang^1^, Shuqin Liu^2^*, Jingui Huang^3^, Yiquan Huang^3^, Chao Chen^3^, Qijun Ruan^3^, Pengran Guo^3^, Gangfeng Ouyang^1,3^*

1. School of Chemical Engineering and Technology, Sun Yat-sen University, Zhuhai 519082, China
2. College of Environment and Climate, Guangdong Provincial Key Laboratory of Environmental Pollution and Health, Jinan University, Guangzhou 511443, China
3. Guangdong Provincial Key Laboratory of Chemical Measurement and Emergency Test Technology, Guangdong Provincial Engineering Research Center for Ambient Mass Spectrometry, Institute of Analysis, Guangdong Academy of Sciences (China National Analytical Center, Guangzhou), Guangzhou 510070, China

*Corresponding author. Email: liushuqin@jnu.edu.cn; cesoygf@mail.sysu.edu.cn

**Section S1 Dissolved organic carbon (DOC) measurement**

DOC concentration was quantified by measuring each sample three times using multi N/C 2100 TOC analyzer (JENA, Germany). To prepare for analysis, all samples were acidified to pH＜2 with 1M HCl solution and stored in the dark until testing. A standard stock solution was created with potassium hydrogen phthalate (KHP, ~ 1 mg C/L). It was used daily to check the measurement precision and accuracy. The relative analytical error was below 2%.

**Section S2 Fluorescence measurements and parallel factor analysis (PARAFAC)**

Three-dimensional excitation emission matrix of dissolved organic matter (DOM) samples was obtained by a fluorescence spectrometer (HORIBA Aqualog, version 3.6, Japan). Each sample was scanned three times. The scan program was set as follows: the emission (Em) wavelengths from 200 to 600 nm with 1-nm steps and increasing excitation (Ex) wavelengths from 250 to 550 nm at 5 nm increment. The integration time was fixed at 0.5 s. The fluorescence responses to ultrapure water were determined as background blank and subsequently subtracted from the measured sample spectra to derive the ultimate EEM dataset. In turn, the primary and secondary Rayleigh and Raman peaks were eliminated and then interpolated, and fluorescence intensities were standardized to Raman units (R.U.), according to the previous methods.^[1, 2]^ PARAFAC analysis was performed in MATLAB R2022b through using the DOMFlour in accordance with previously reported tutorial.^[3, 4]^ The peak fluorescence intensity for the individual component was represented by the maximum fluorescence intensities (*F*_max_).

For PE-derived DOM, there were clearly recognizable fluorescence signatures in Figure S2. Fluorescence peaks detected in the leached DOM from black and white PE were only observed in the fluorescence EEM region with Em < 380 nm, but the DOM released from red and yellow PE exhibited additional peaks in the humic-like fluorescence region, which spanned from 380 to 550 nm (Em). A four-component PARAFAC model further described the variations of these fluorescence features (Figure S3, Table S1). Component 1 (C1), Component 3 (C3) and Component 4 (C4) each had an individual fluorescence peak at Ex_max_ (maximum of the excitation wavelength) / Em_max_ (maximum of the emission wavelength) for 290 / 405 nm, 270 / 310 nm and 320 / 430 nm. Component 2 (C2) has one maximum emission peak and two maximum excitation peaks 270 (295) / 365 nm (Ex_max_ / Em_max_). According to the previous studies,^[5, 6]^ C1 and C4 corresponded to a structurally complex humic acid component, while C2 and C3 were classified as the commonly reported protein/phenol-like compounds. Red PE derived DOM contained large amounts of C1 with contributing 65.1% of *F_max_* values, suggesting that humic-like substances were the main DOM components released by red PE. C2 was the dominant component in the DOM leached from white (39.3%) and yellow PE (45.5%). C3 occupied the largest proportion in the DOM leached from black and white PE, with a *F_max_* contribution of 93.4% and 57.9%, respectively. On the contrary, the *F_max_* values of C4 were the lowest but were higher in those from red and yellow PE.

For DOM extracted from lake water samples, EEM-PARAFAC analysis identified five fluorescent components from all EEM dataset (Figure S15 andS16, Table S4). C1 (Ex_max_ / Em_max_ = ＜250 (330) / 425 nm) was considered to be widely existing terrestrial humic-like substance.^[7]^ C2 (Ex_max_ / Em_max_ = 295 / 410 nm) was assigned as microbially humic-like substances.^[8]^ The lake samples added different PE-derived DOM all contained a large number of these two humic-like fluorescence substances with a *F_max_* contribution of more than 50%. C3 (Ex_max_ / Em_max_ = 295 / 365 nm) was taken for microbially microbial tryptophane-like components.^[9]^ C4, with an excitation maximum at 270 nm and emission maximums at 330 nm and 520 nm, was classified as microbial tryptophan-like and fulvic-like substances.^[10]^ They had a low relative abundance in the treatment enriched with red PE-derived DOM. Component 5 (C5) accounted for a low proportion in all DOM samples whether at the initial stage of incubation or after seven days incubation. It showed double excitation (330 nm and 365 nm) and double emission (470 nm and 520 nm) peaks, exhibiting a high mixed degree of unknown humic-like substances.^[11]^

**Section S3 Solid phase extraction (SPE) process and Fourier transform ion cyclotron resonance mass spectrometry (FT-ICR MS) analysis**

DOM extractions were performed using SPE procedure with Bond Elute PPL cartridges (3 mL, 200 mg, Agilent). Briefly, the cartridges were activated with 30 mL of HPLC-grade methanol, 30 mL of acidified distilled water and 30 mL of distilled water. All DOM samples were acidified to pH 2 with HCl (1 M) and passed through the cartridges by gravity within 1.5 hours. The cartridges were then rinsed in sequence with 30 mL of acidified distilled water (pH 2) and 30 mL distilled water, finally dried under nitrogen. All cartridges were eluted with 3 mL of methanol to obtain the final eluted DOM extracts. Solvent blanks and PPL extraction blanks were prepared and analyzed to check for possible contamination and perform blank correction.

DOM extract was diluted with distilled water at 1:1 volume ratio for FT-ICR MS analysis. Samples were injected into the ESI source at a rate of 120 μL/h, with a needle voltage of -3.8 kV. Mass spectra were collected over 300 scans, with an ion accumulation time of 0.6 s in a hexapole ion trap and the mass-to-charge ratio (M/Z) range of 100-1000 m/z. The instrument was calibrated with 10 mM sodium formate solution before sample analysis, and the internal standard ion m/z 311.1686 was determined for on-line calibration in the data gathering process. After sample analyses, the acquired raw MS data were processed using Bruker DataAnalysis software (version 5.0). Firstly, the raw spectra of each sample were converted to a list of m/z values with a signal-to-noise threshold of 6 and a default intensity threshold of 100. The mass error for a given chemical formula between the measured mass and the theoretical mass was less than 1 ppm. Next, the measured spectra were internally calibrated using a known homologous series of natural organic matter compositions.

**Section S4 Additional details of FT-ICR MS data processing**

The van Krevelen diagram was conducted using the ratios of oxygen to carbon (O/C) and hydrogen to carbon (H/C), and were delineated by O/C and H/C ratios into seven categories: saturated compounds (O/C = 0 – 0.52, H/C = 1.5 – 2.2), aminosugars (O/C = 0.52 – 0.71, H/C = 1.5 – 2.2), carbohydrates (O/C = 0.71 – 1.2, H/C = 1.5 – 2.4), tannins (O/C = 0.67–1.2, H/C = 0.52 – 1.5), lignins (O/C = 0.1 – 0.67, H/C = 0.7 – 1.5), unsaturated hydrocarbons (O/C = 0 – 0.1, H/C = 0.7 – 1.5), and condensed aromatic structures (O/C = 0 – 0.67, H/C = 0.2 – 0.7). Some general molecular traits were calculated in our work.^[12, 13]^ The modified index (AI_Mod_) was calculated from the formulas to estimate fraction of aromatic and condensed aromatic groups. Nominal oxidation state of carbon (NOSC) indicates the average oxidation state of all carbons per formula independent of the chemical structure. Double bond equivalence (DBE) represents the number of unsaturated bonds and rings in a molecule. Intensity-weighted averaged (wa) values were calculated on the basis of the ratio of each compound intensity by the sum of the intensity values which described elsewhere.^[14]^

$${AI}_{Mod}=1+\frac{C-0.5C-S-0.5\left( N+H \right)}{C-0.5O-S-N} (1)$$

$$NOSC=4-\frac{4C+H-3N-2O-2S}{C} (2)$$

$$DBE=1+\frac{1}{2}\left( 2C-H+N \right) (3)$$

Where C, H, O, N, and S refer to the number of atoms per formula of carbon, hydrogen, nitrogen, oxygen, and sulfur, respectively.

**Section S5 DNA extraction and DNA library construction**

Total DNA was extracted from biomass-containing filters using CTAB method, and their concentration and quality were further determined by 1% agarose gel electrophoresis. The obtained DNA samples were stored at - 80 °C for the next library construction.

Sequencing libraries were generated using NEBNext Ultra DNA Library Prep Kit for Illumina (NEB, USA) following manufacturer’s recommendations. Briefly, DNA extract was fragmented to a size of 350 bp, and in turn, the obtained DNA fragments were ligated with index barcode sequences, end-polished, A-tailed and ligated with the full-length adaptor. After PCR amplification, library quality was assessed on the Agilent 5400 system (Agilent, USA) and quantified by real-time PCR. Finally, the qualified libraries were pooled and sequenced on Illumina platforms according to effective library concentration and required data amount.

**Section S6 16S rRNA gene metabarcoding sequencing and metagenomic sequencing**

Bacterial community composition was characterized by 16S rRNA gene metabarcoding (V3–V4) on an Illumina NovaSeq platform using paired-end 2×250 bp. Community DNA was PCR-amplified utilizing primers F314 (5’-CCTAYGGGRBGCASCAG-3’) and R806 (5’-GGACTACNNGGGTATCTAAT-3’) targeting the V3−V4 region of the 16S rRNA gene. PCR reactions (25 μL) contained 15 μL Phusion® High-Fidelity PCR Master Mix (New England Biolabs), 2 μM forward/reverse primers, and ~10 ng template DNA. Thermal cycling was according to the manufacturer’s recommendations. Then, PCR products were mixed with an equal volume of 1×TAE buffer and analyzed via 2% agarose gel electrophoresis, and further were purified in equimolar concentrations using the Universal DNA Kit (TianGen, China). On the other hand, metagenomic sequencing was synchronously performed on the Illumina platform with PE150 strategy.

**Section S7 Bioinformatics analysis**

Raw sequence data of 16S rRNA gene metabarcoding were processed in the QIIME2 program following the recommended tutorials (https://docs.qiime2.org/2019.1/). Briefly, sequence demultiplexing, merging, filtering, denoising, and chimera checking were performed before obtaining operational taxonomic units (OTUs). We then assigned taxonomy with Greengenes2 Database and removed the OTUs belonging to chloroplasts or not assigned to bacteria or archaea. OTUs were normalized to the total number of reads obtained for each sample to obtain their relative abundance. The raw sequencing data have been deposited in the NCBI Sequence Read Archive (SRA) under accession number PRJNA1336635.

The raw metagenomic data were also qualified by FastQC, and then trimmed using Trimmomatic to remove adaptors (≥ 15 bp sequence overlap with adapter sequence) and low-quality reads (with length less than 150 bp or with quality score below 30). After the above filtration, all the raw metagenomic datasets were normalized to 28702744 clean reads for each sample to achieved for further ARGs annotation in CARD database. The raw sequencing data have been deposited in the NCBI Sequence Read Archive (SRA) under accession number PRJNA1336967.

**Supplementary Figures**

**
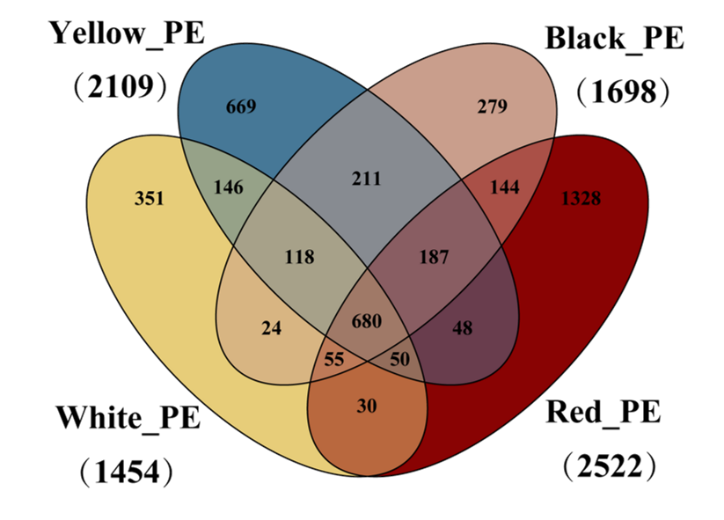
**

**Figure S1** The molecular richness of DOM released from four colored PE shopping bags via Venn diagram, which was expressed as the total number of DOM molecular formulas identified by FT-ICR MS.


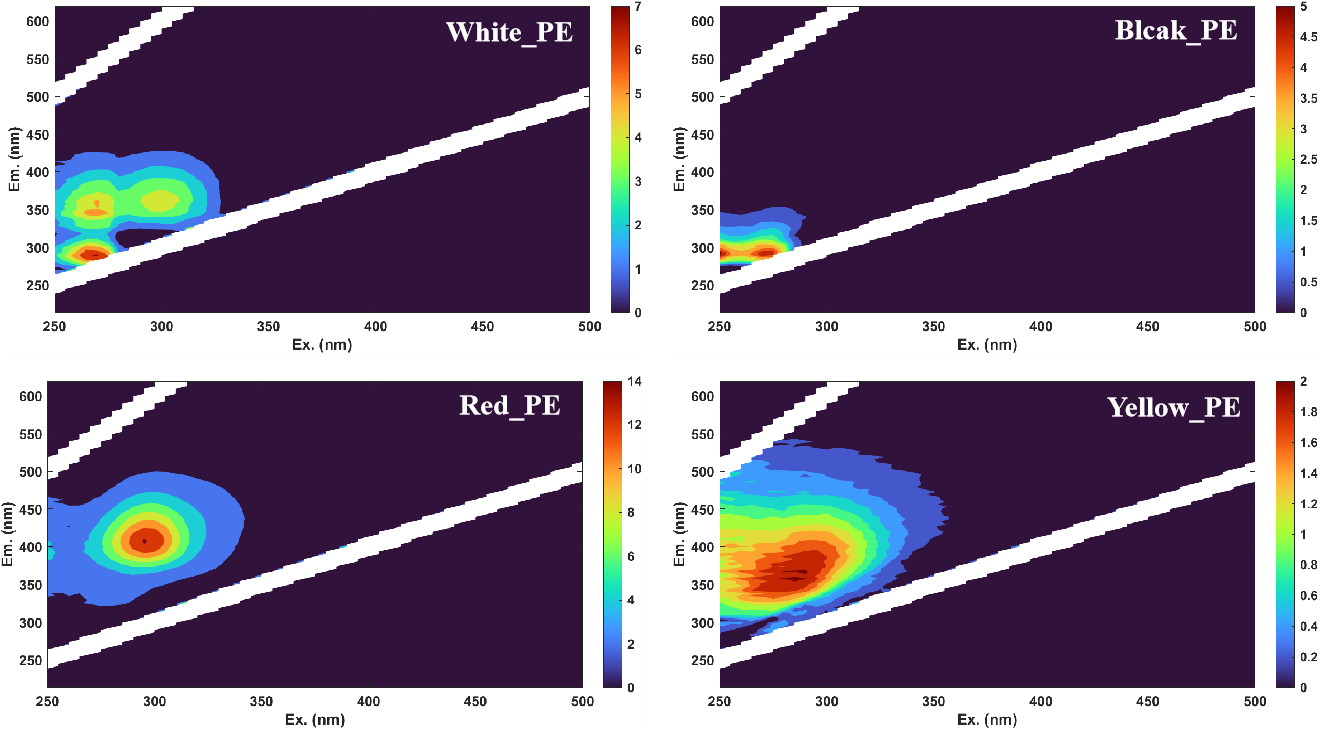


**Figure S2** Fluorescence EEM plots of four PE-derived DOM. EEMs were normalized to the area of the Raman Scattering peak of water at 350 nm excitation to convert fluorescence intensities to Raman Units. Rayleigh and Raman scatters were also eliminated and interpolated.


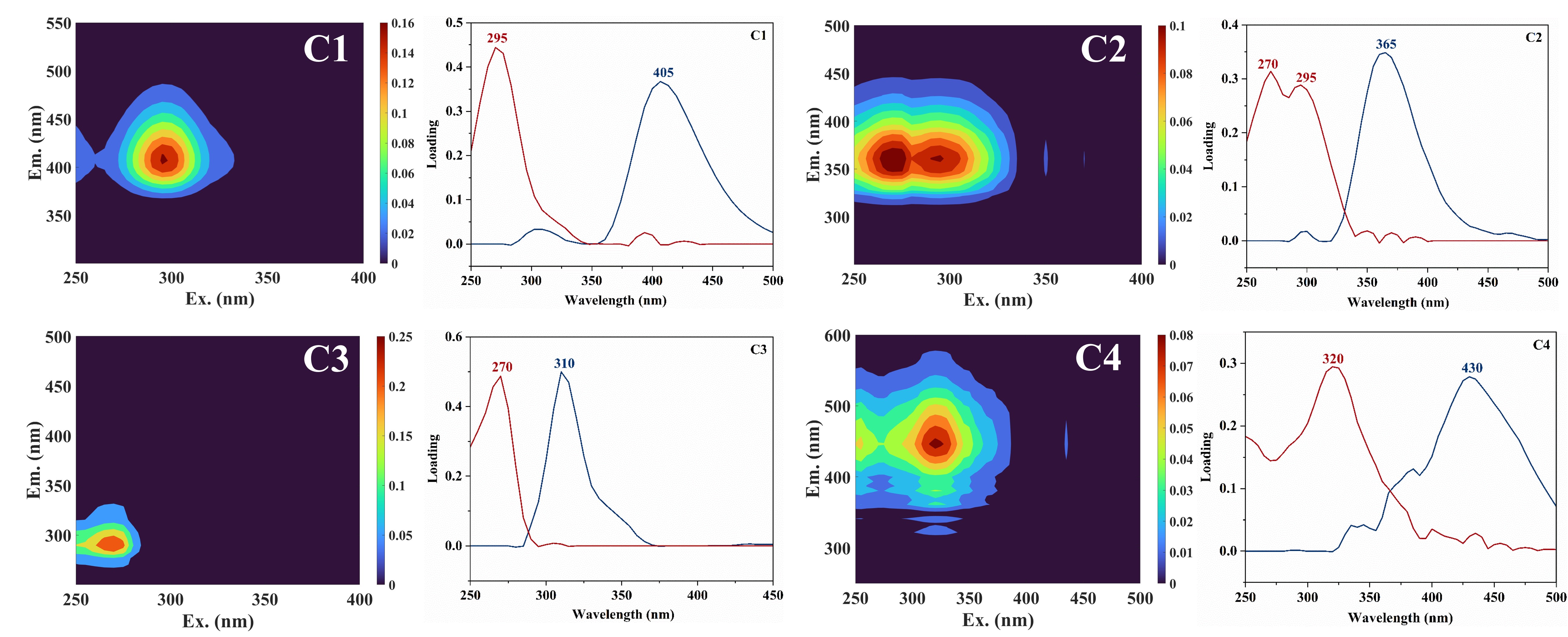


**Figure S3** Four fluorescent components (C1-C4) were identified from the fluorescence spectra of PE-derived DOM samples via PARAFAC analysis.

**
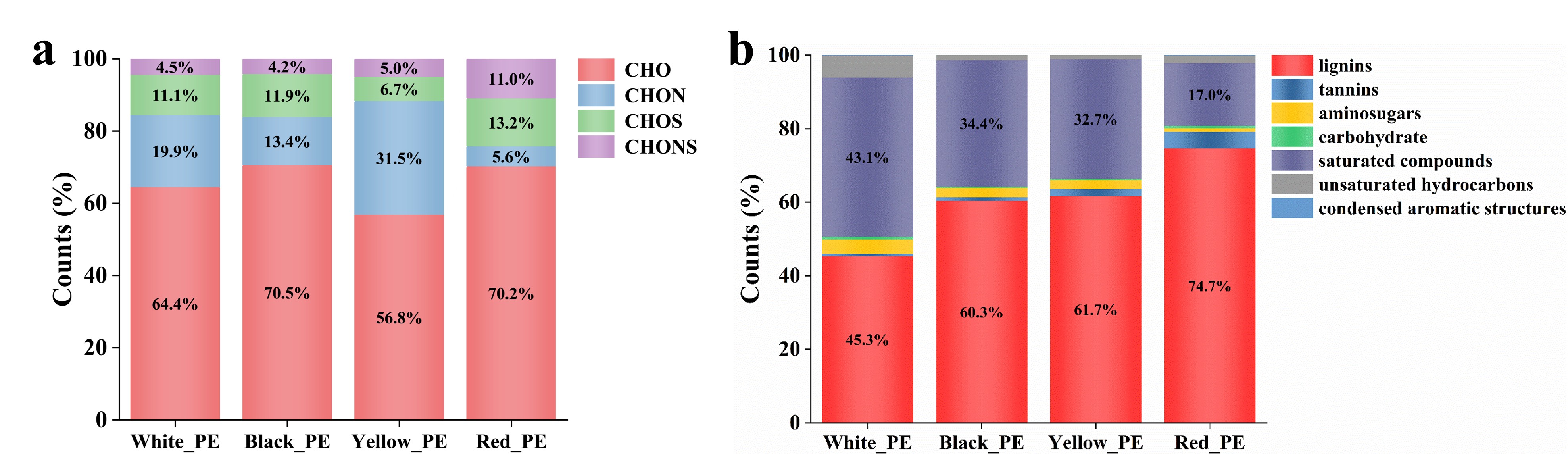
**

**Figure S4** The relative amount of molecule formulas assigned to elemental compositions (a) and compound classes (b) within each colored PE-derived DOM.


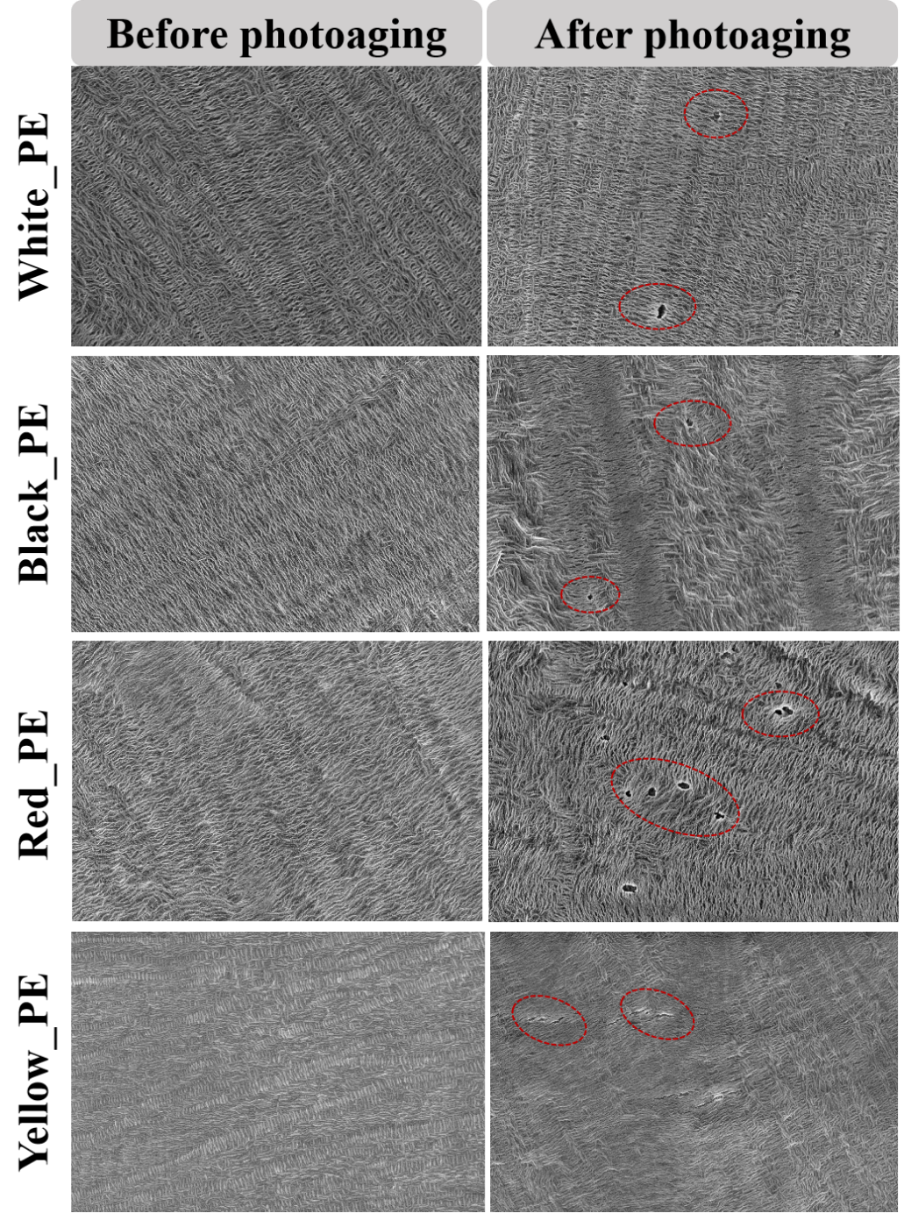


**Figure S5** SEM images showed surface morphological changes of different colored PE plastics before and after ultraviolet irradiation.

**
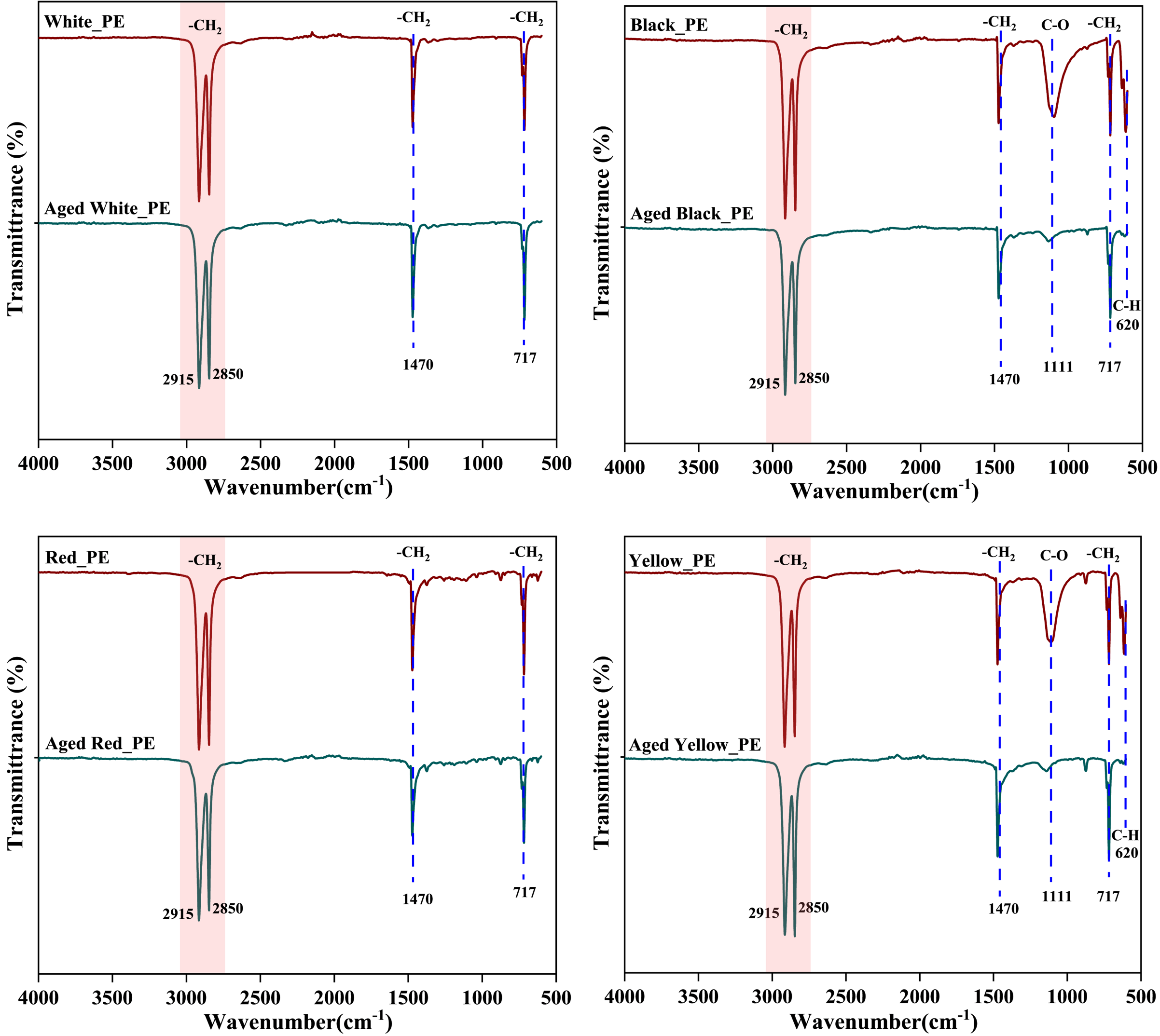
**

**Figure S6** FTIR spectra of different colored PE plastics before (red line) and after (green line) photodegradation under ultraviolet irradiation.


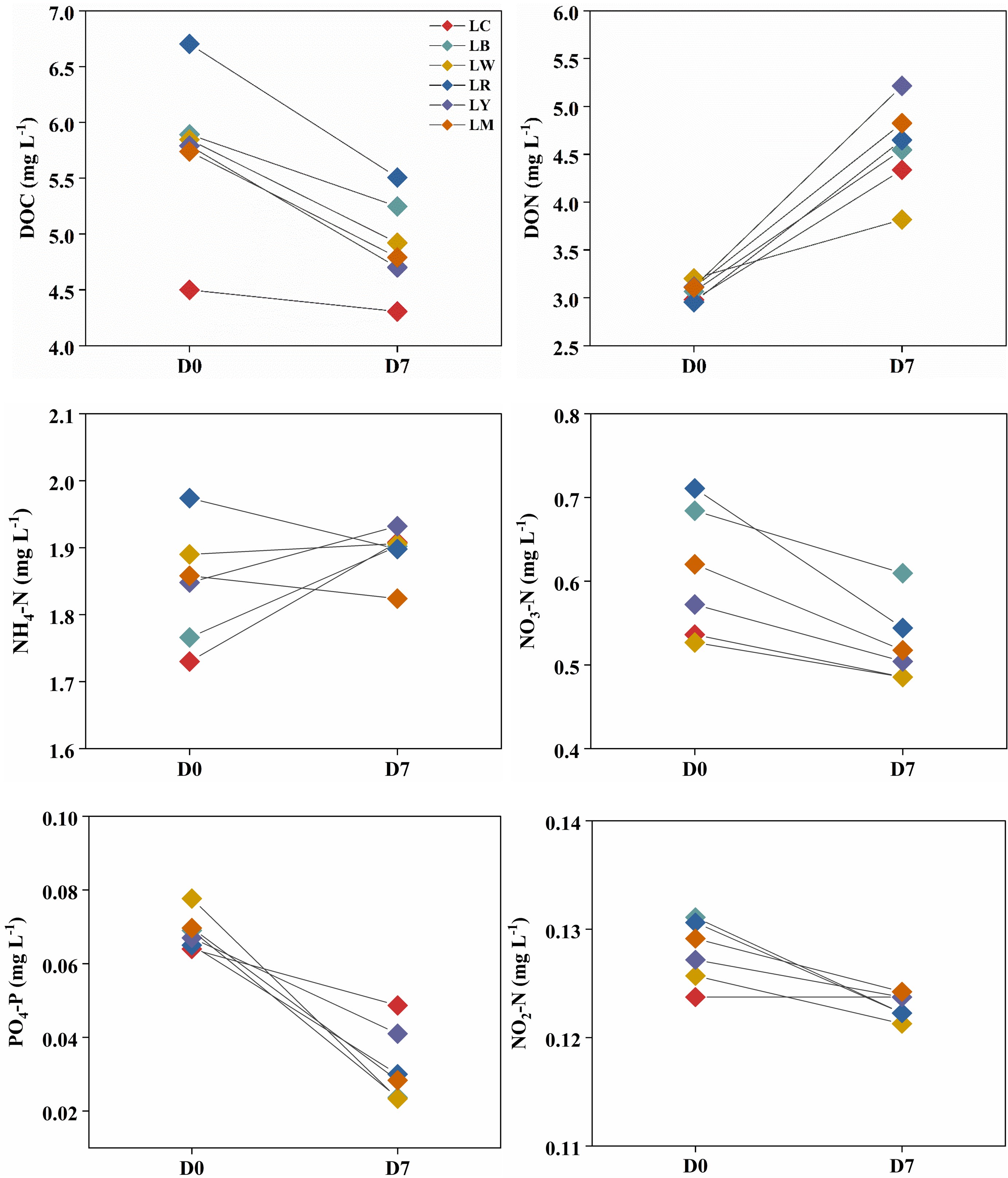


**Figure S7** Bacterial growth caused the quick consumption of available nutrients and the production of DON from day 0 to day 7. Abundance values were reported as the mean (n = 3). Available nutrients included DOC, inorganic nitrogen NH_4_^+^, NO_3_^−^, NO_2_^−^, and phosphate PO_4_^3−^. LC represented as the normal lake water; LB, LW, LR and LY respectively expressed as the lake water added DOM filtration derived from black, white, red and yellow PE; LM was set as a combined treatment, converging four collected PE-derived DOM filtration.

**
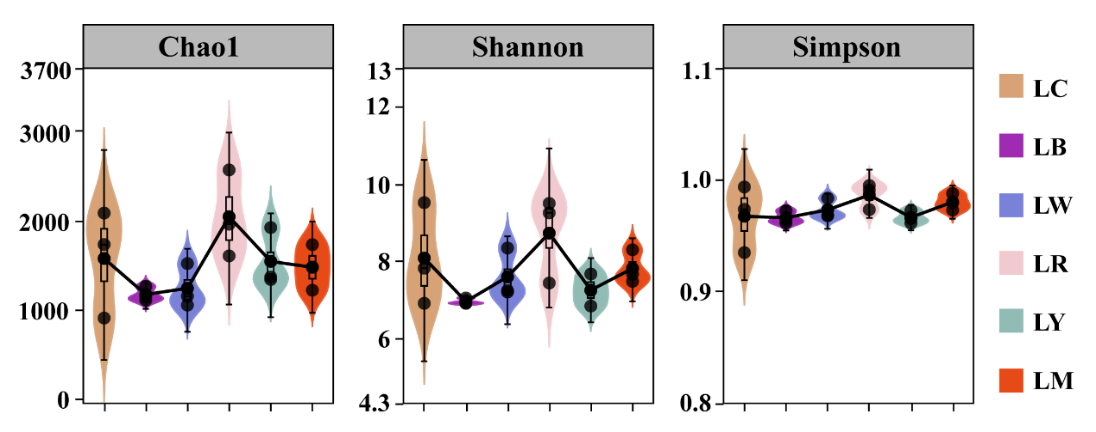
**

**Figure S8** Chao1, Shannon and Simpson indexes were calculated to evaluate bacterial α-diversity (n = 3). Statistical comparison among multiple treatments displayed no significance (*p* > 0.05; One-way ANOVA). LC represented as the normal lake water; LB, LW, LR and LY respectively expressed as the lake water added DOM filtration derived from black, white, red and yellow PE; LM was set as a combined treatment, converging four collected PE-derived DOM filtration.

**
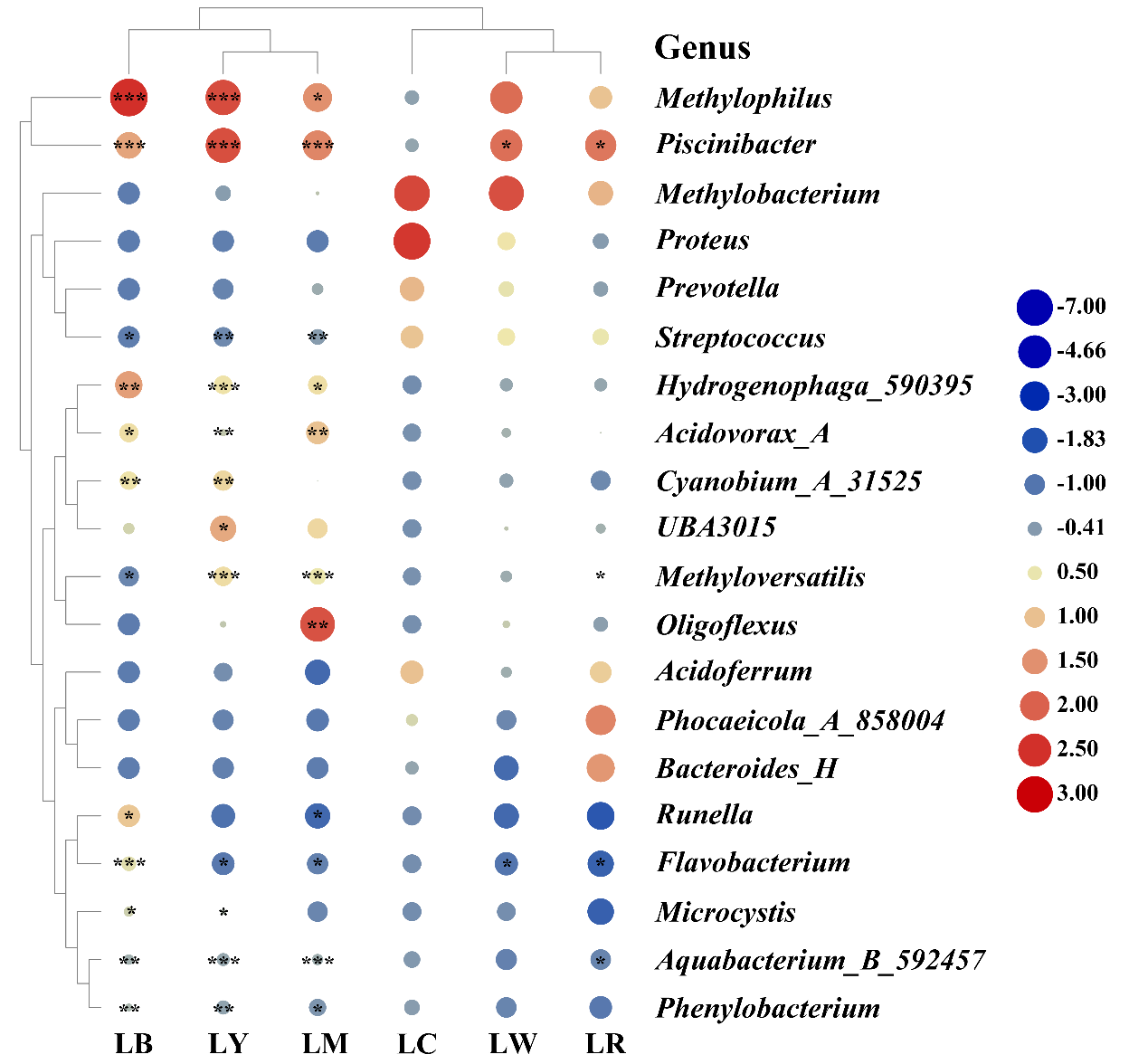
**

**Figure S9** Differentially abundant bacterial genera (top 20) across treatments based on 16S rRNA gene metabarcoding, with the values indicating the relative abundance by log 2 transformed. Abundance values were reported as the mean (n = 3). Asterisks mark statistically significant differences (**p* < 0.05, ** *p* < 0.01, *** *p* < 0.001; Student’s t-test).

**
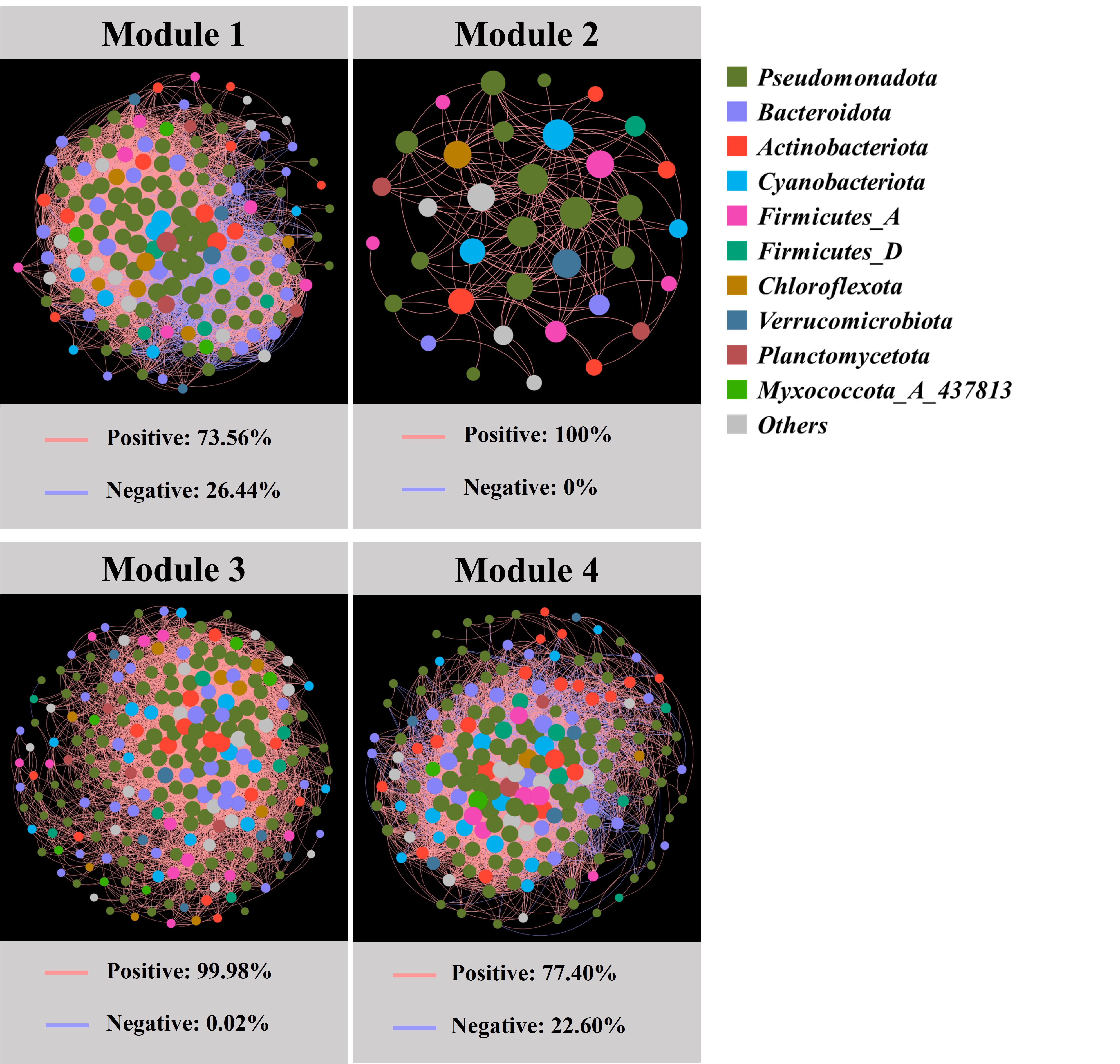
**

**Figure S10** Four ecological clusters were obtained from bacterial co-occurrence network, dominated by positive associations. Nodes were colored based on taxonomy at the phylum level, and node size was defined in degrees, with larger nodes reflecting higher degree values. A link indicated a strong and significant (|*r*| > 0.7 and *p* < 0.01) correlation, with red edges indicating positive associations and purple edges indicating negative correlations.

**
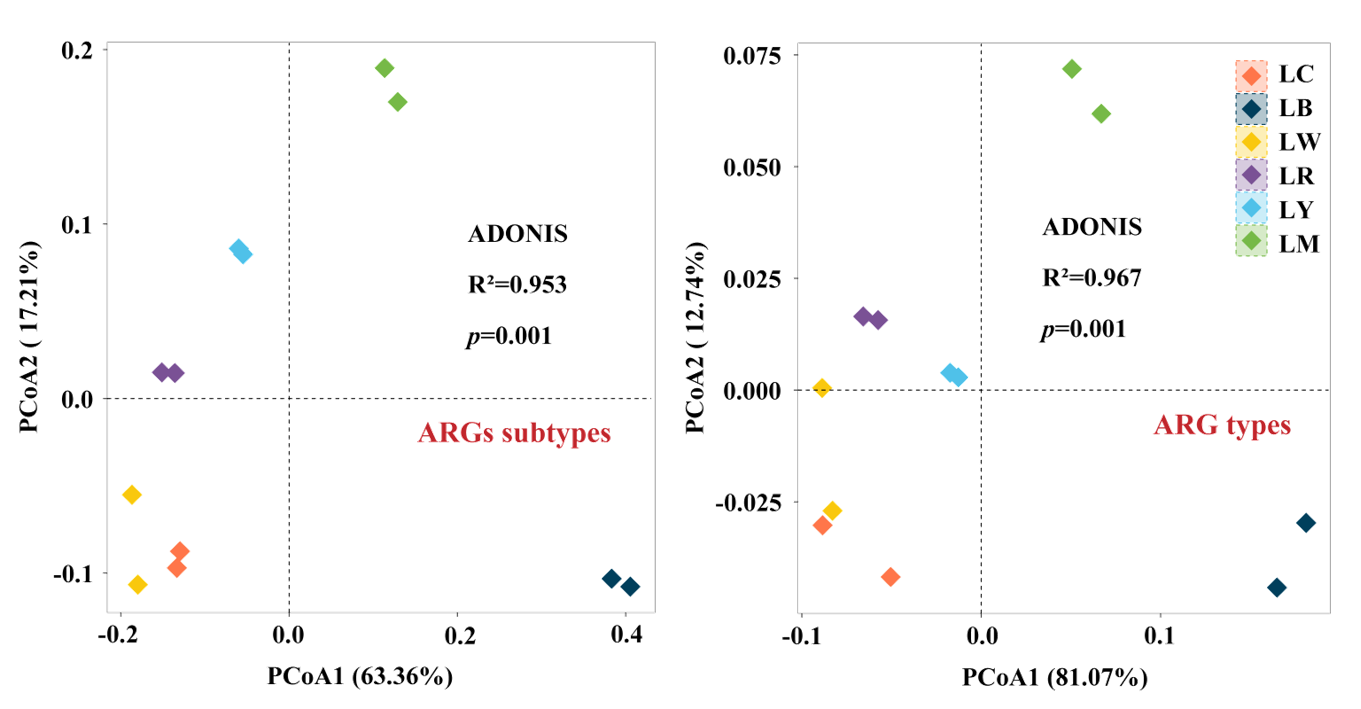
**

**Figure S11** PCoA ordination depicted the distributing patterns of ARG types and ARG subtypes, and showed a clear separation among treatments. Adonis tests were conducted between ARG community compositions grouped by types and subtypes using R package vegan.

**
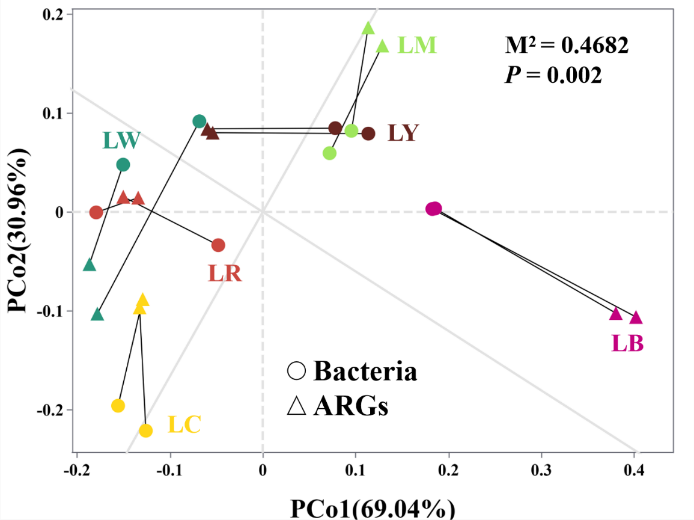
**

**Figure S12** Procrustes test of the significant correlation between ARG profiles and bacterial community composition based on Bray-Curtis distances. LC represented as the normal lake water; LB, LW, LR and LY respectively expressed as the lake water added DOM filtration derived from black, white, red and yellow PE; LM was set as a combined treatment, converging four collected PE-derived DOM filtration.


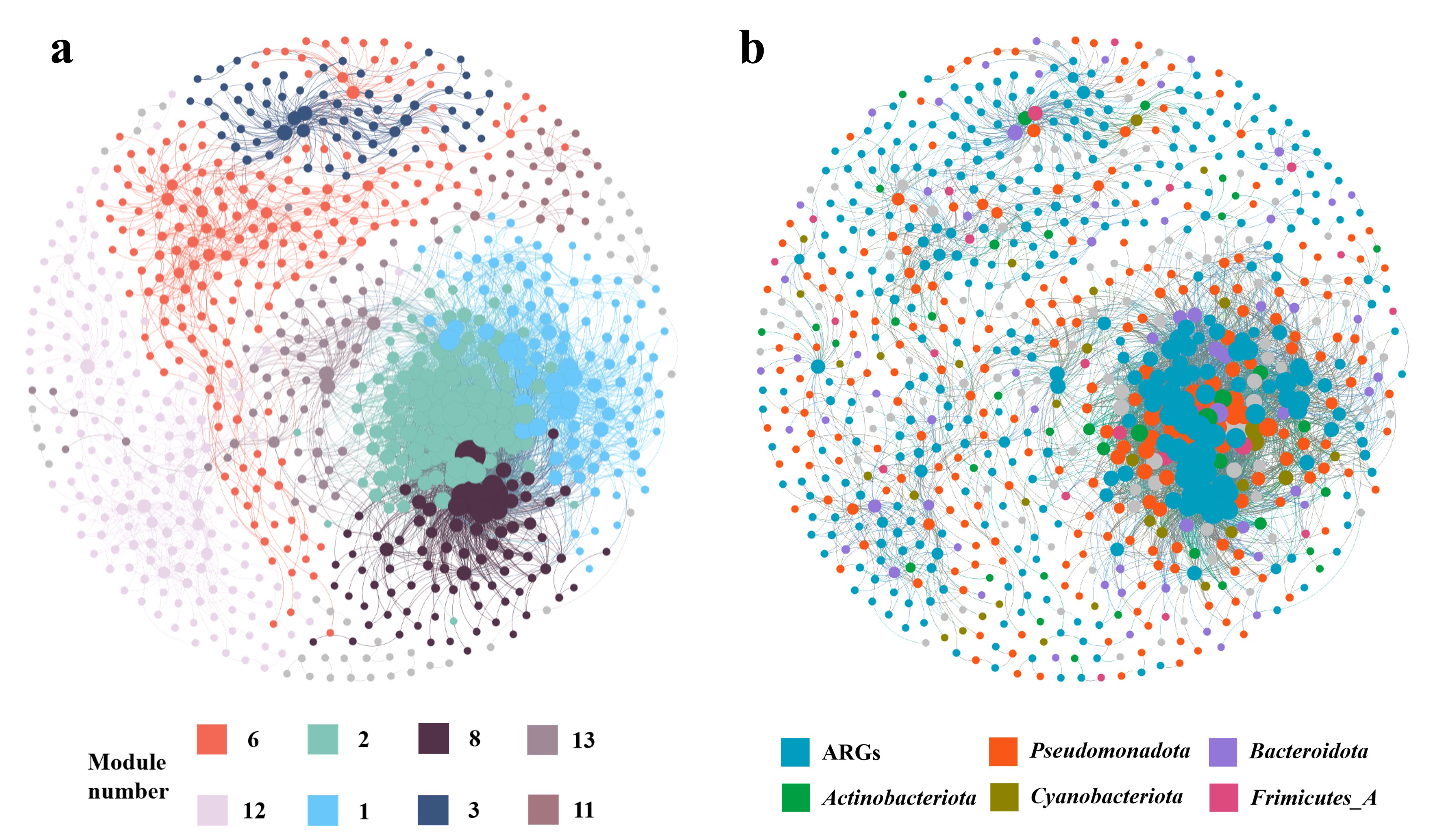


**Figure S13** Network constructed upon significant positive correlations between dominant ARG subtypes and bacterial OTUs, which were colored with respect to modularity cluster (a) and according to ARG subtypes and bacterial phyla (b). A connection represents a strong (Spearman’s correlation coefficient ρ > 0.7) and significant (*p* <0.01) correlation. The size of each node is proportional to the number of connections. The network exhibited a high degree of modularity (0.467), and 94.4 % of vertices were accounted for by only eight of the twenty-four total modules (a). Bacterial phyla *Pseudomonadota*, *Bacteroidota*, *Actinobacteriota*, *Cyanobacteriota*, and *Firmicutes_A* could explain 80.1 % of the potential hosts (b).

**
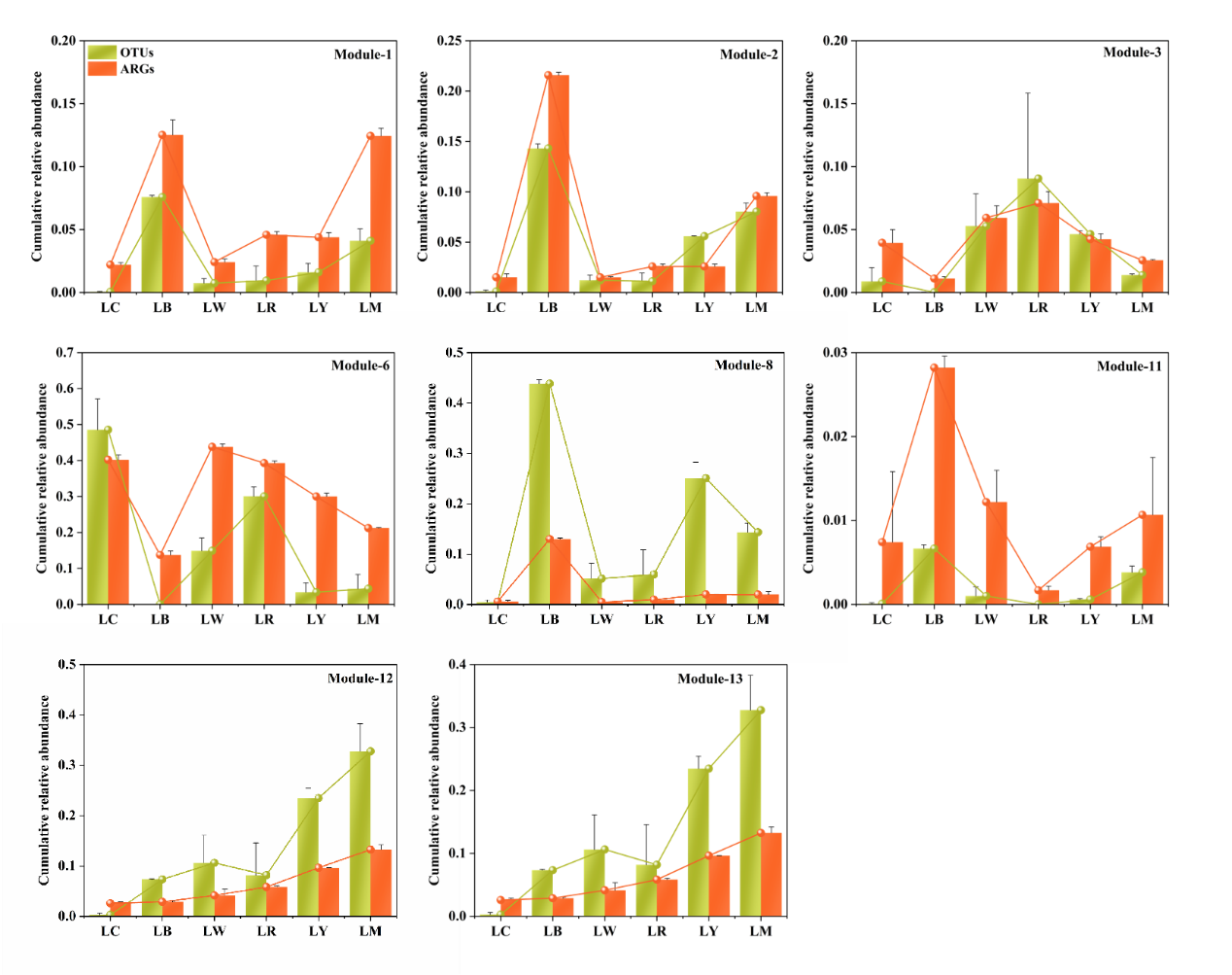
**

**Figure S14** Abundance trends of ARGs and their potential hosts within modularity clusters under different treatments. LC represented as the normal lake water; LB, LW, LR and LY respectively expressed as the lake water added DOM filtration derived from black, white, red and yellow PE; LM was set as a combined treatment, converging four collected PE-derived DOM filtration.

**
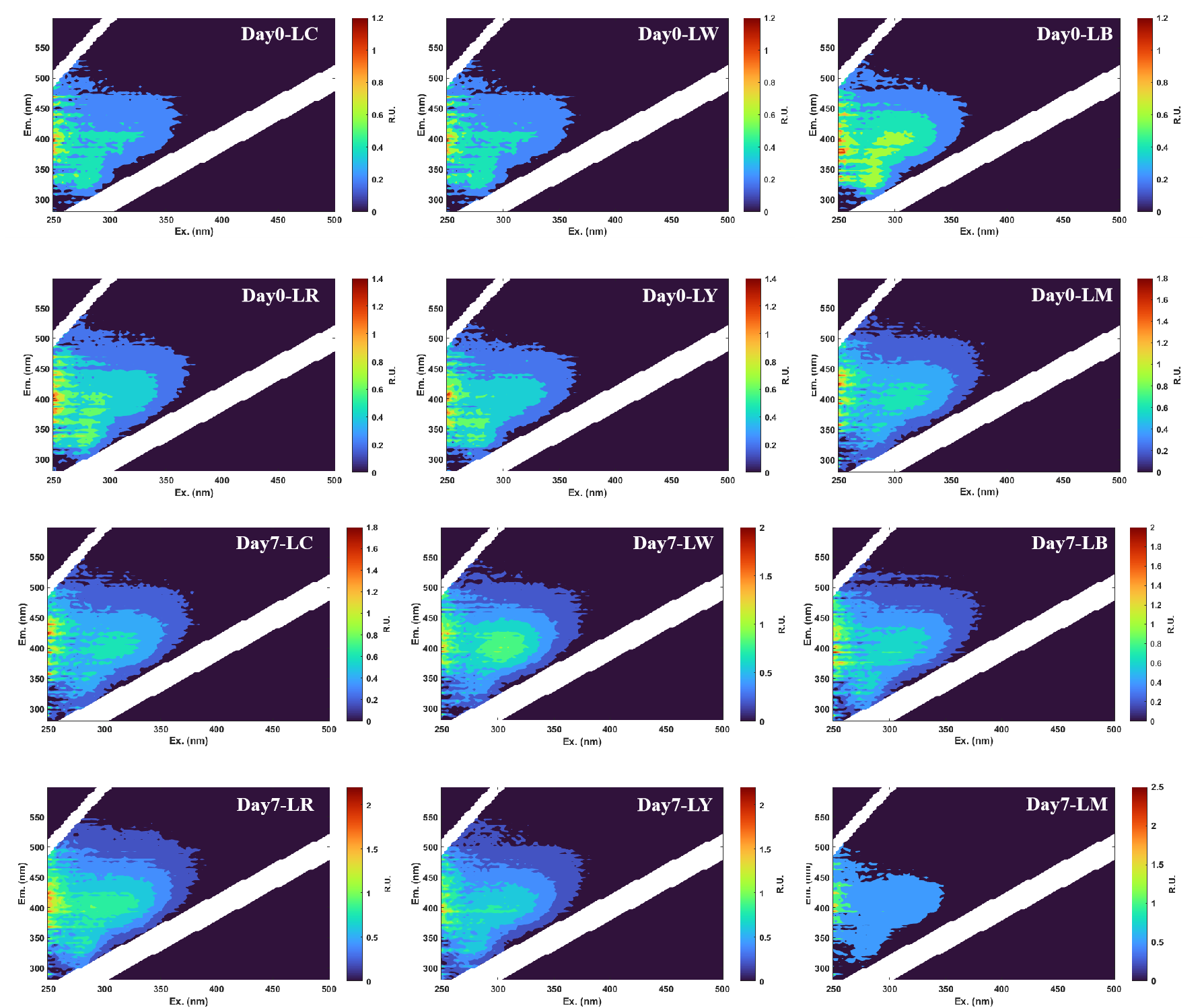
**

**Figure S15** Fluorescence EEM plots of lake water DOM samples in day 0 and day 7. EEMs were normalized to the area of the Raman Scattering peak of water at 350 nm excitation to convert fluorescence intensities to Raman Units. Rayleigh and Raman scatters were also eliminated and interpolated. LC represented as the normal lake water; LB, LW, LR and LY respectively expressed as the lake water added DOM filtration derived from black, white, red and yellow PE; LM was set as a combined treatment, converging four collected PE-derived DOM filtration.

**
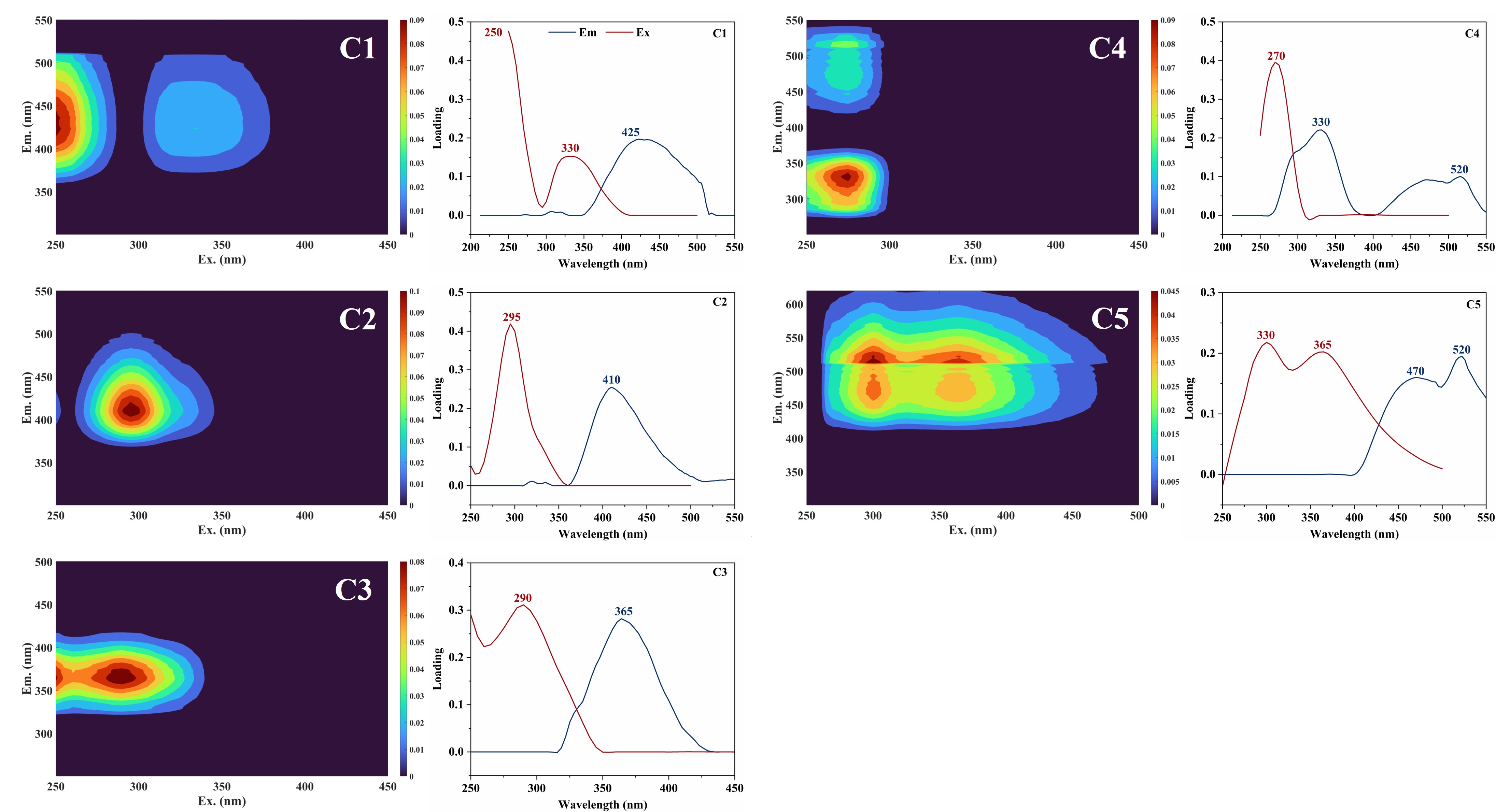
**

**Figure S16** Spectral properties of five fluorophores (C1, C2, C3, C4, and C5) identified by PARAFAC analysis, based on the EEM spectral data from lake water DOM samples collected in the microcosm experiments.

**
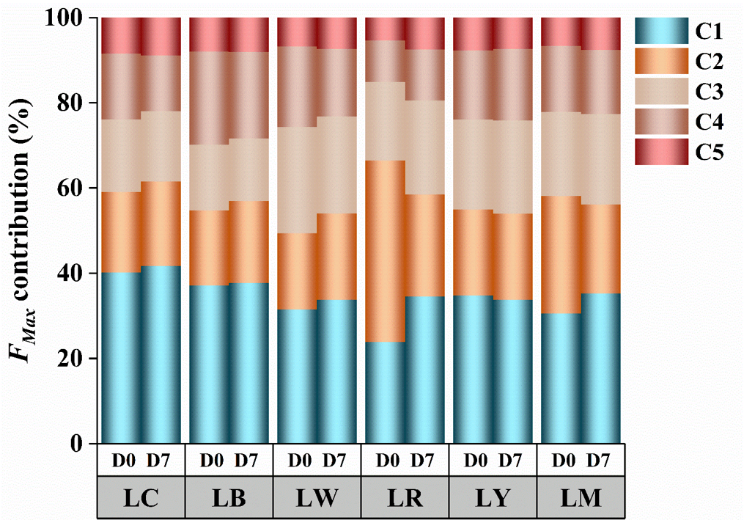
**

**Figure S17** Changes of five fluorescent components obtained from lake water DOM samples before and after microcosm experiments. LC represented as the normal lake water. LB, LW, LR and LY respectively expressed as the lake water added DOM filtration derived from black, white, red and yellow PE; LM was set as a combined treatment, converging four collected PE-derived DOM filtration.

**
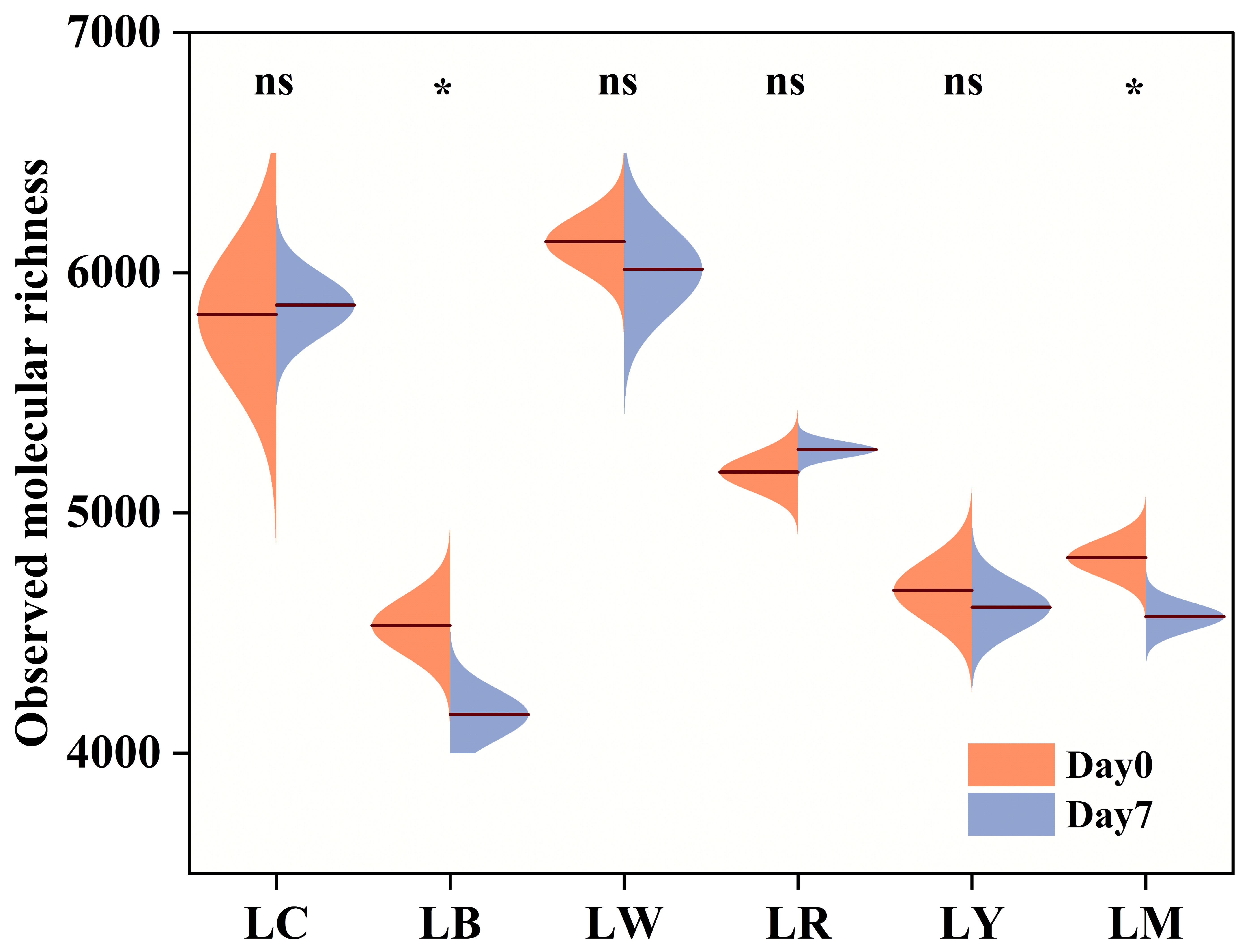
**

**Figure S18** Comparison in DOM molecule richness before and after microcosm experiments (**p* < 0.05; ns, no significant difference; Student’s t-test). LC represented as the normal lake water. LB, LW, LR and LY respectively expressed as the lake water added DOM filtration derived from black, white, red and yellow PE; LM was set as a combined treatment, converging four collected PE-derived DOM filtration.

**
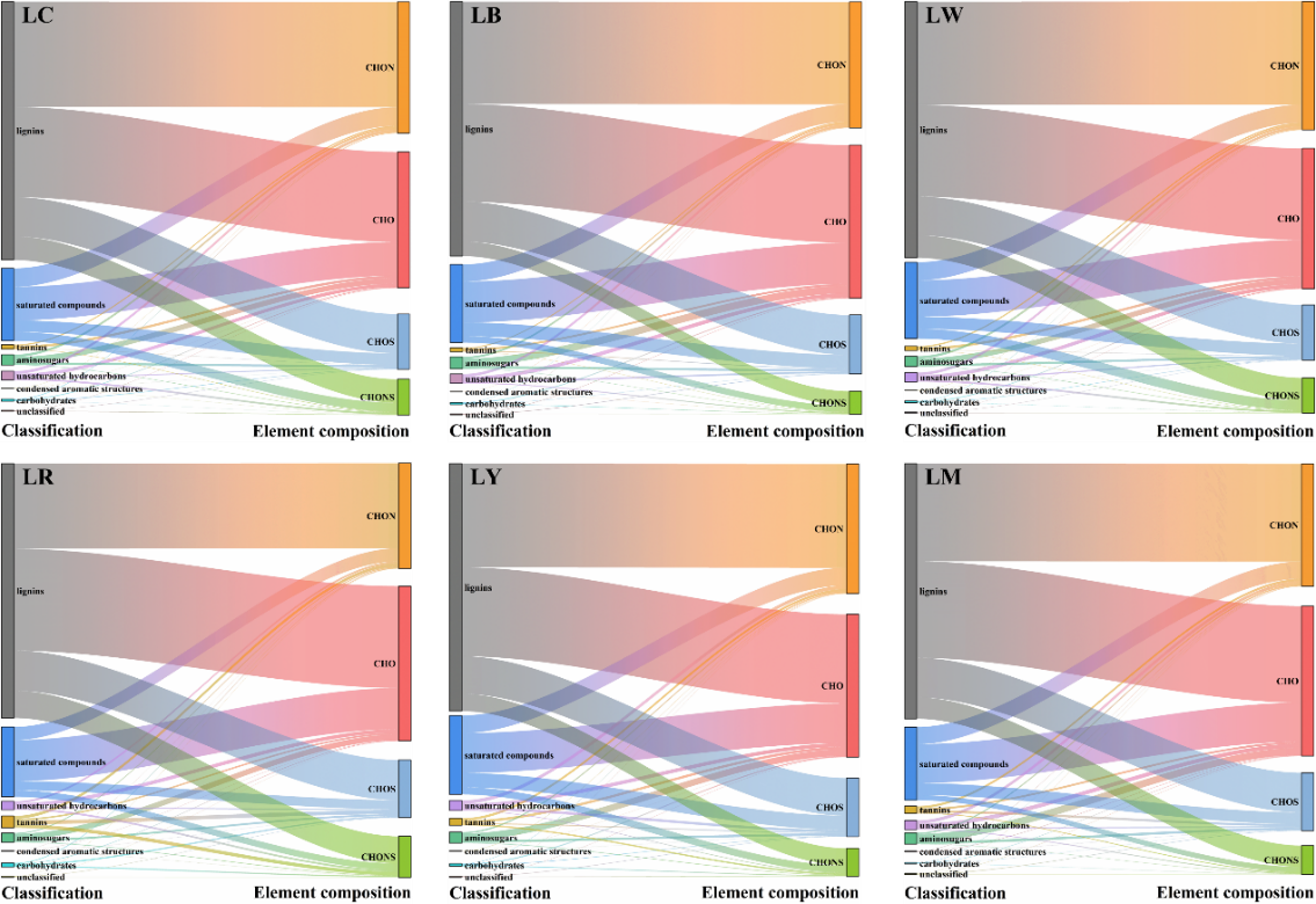
**

**Figure S19** Classification and element composition of DOM formulas detected in per treatment at the end of microcosm experiments and their contrast relation. LC represented as the normal lake water; LB, LW, LR and LY respectively expressed as the lake water added DOM filtration derived from black, white, red and yellow PE; LM was set as a combined treatment, converging four collected PE-derived DOM filtration.

**
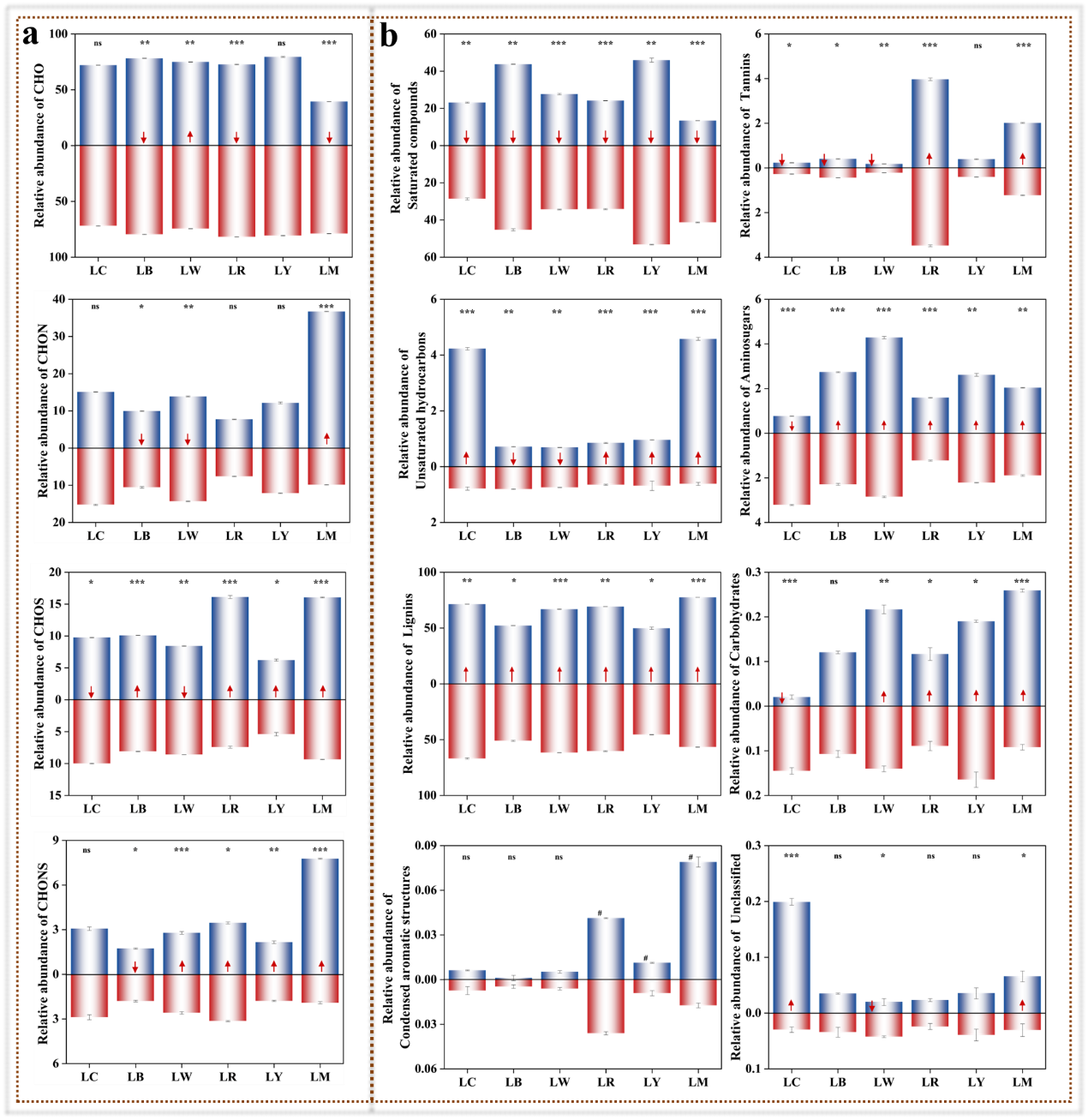
**

**Figure S20** Changes on relative abundance of elemental composition (a) and classification of DOM molecules (b) after microcosm experiments (**p* < 0.05, ** *p* < 0.01, *** *p* < 0.001; ns, no significant difference; Student’s t-test; # denoted that both groups showed zero standard deviation, making the t-test inapplicable). Upper and lower bars represented the relative peak intensity of DOM compounds in day 7 and day 0, respectively. LC represented as the normal lake water; LB, LW, LR and LY respectively expressed as the lake water added DOM filtration derived from black, white, red and yellow PE; LM was set as a combined treatment, converging four collected PE-derived DOM filtration.

**
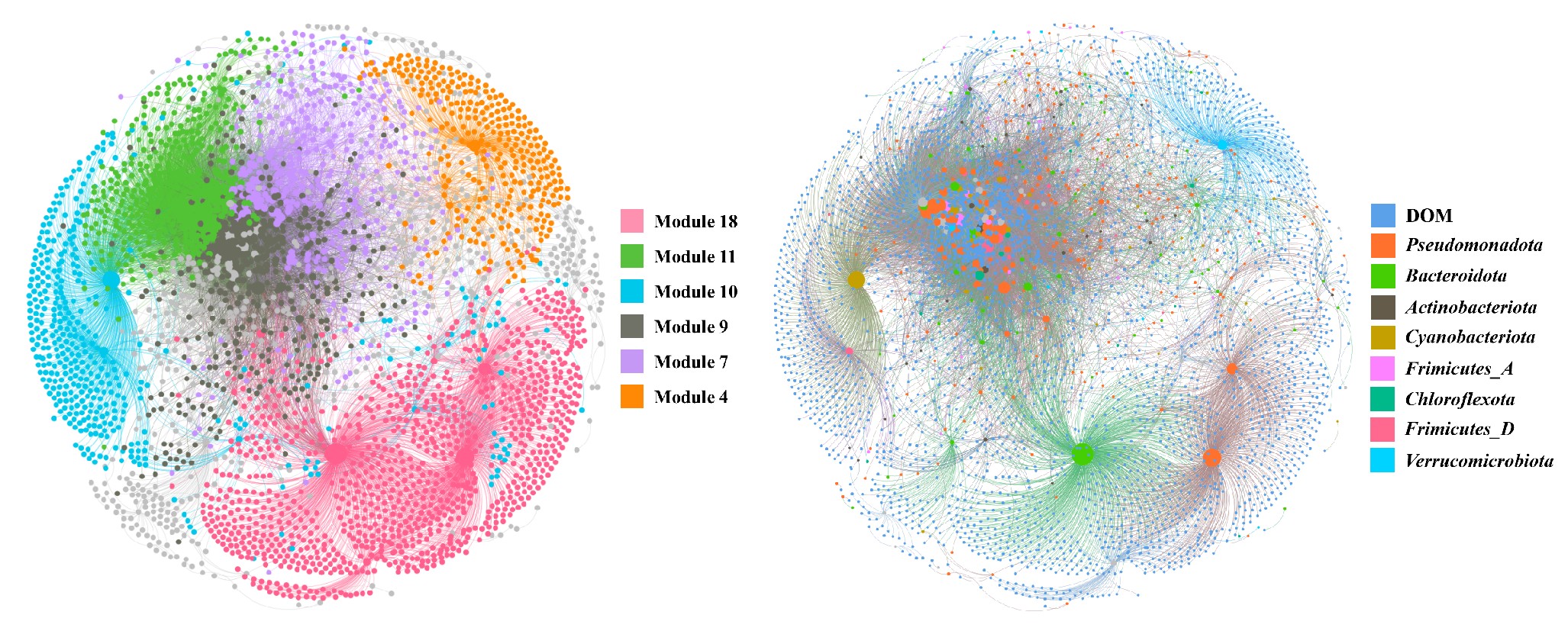
**

**Figure S21** Bacterial interactions with DOM molecules (i.e., Bacteria-DOM associations). Spearman correlation analysis showed that there was a total of 460 bacterial OTUs, whose relative abundances exhibited strong correlations (|*r*| > 0.7 and *p* < 0.01) with the relative intensities of 2826 DOM molecules. The network was with modularity value 0.406, and the top six of the twenty-five total modules explained 85.7% of all vertices, as colored in the network diagram (a). For clarity, the top eight bacterial phyla strongly related to DOM molecules in the network were also colored (b).

**
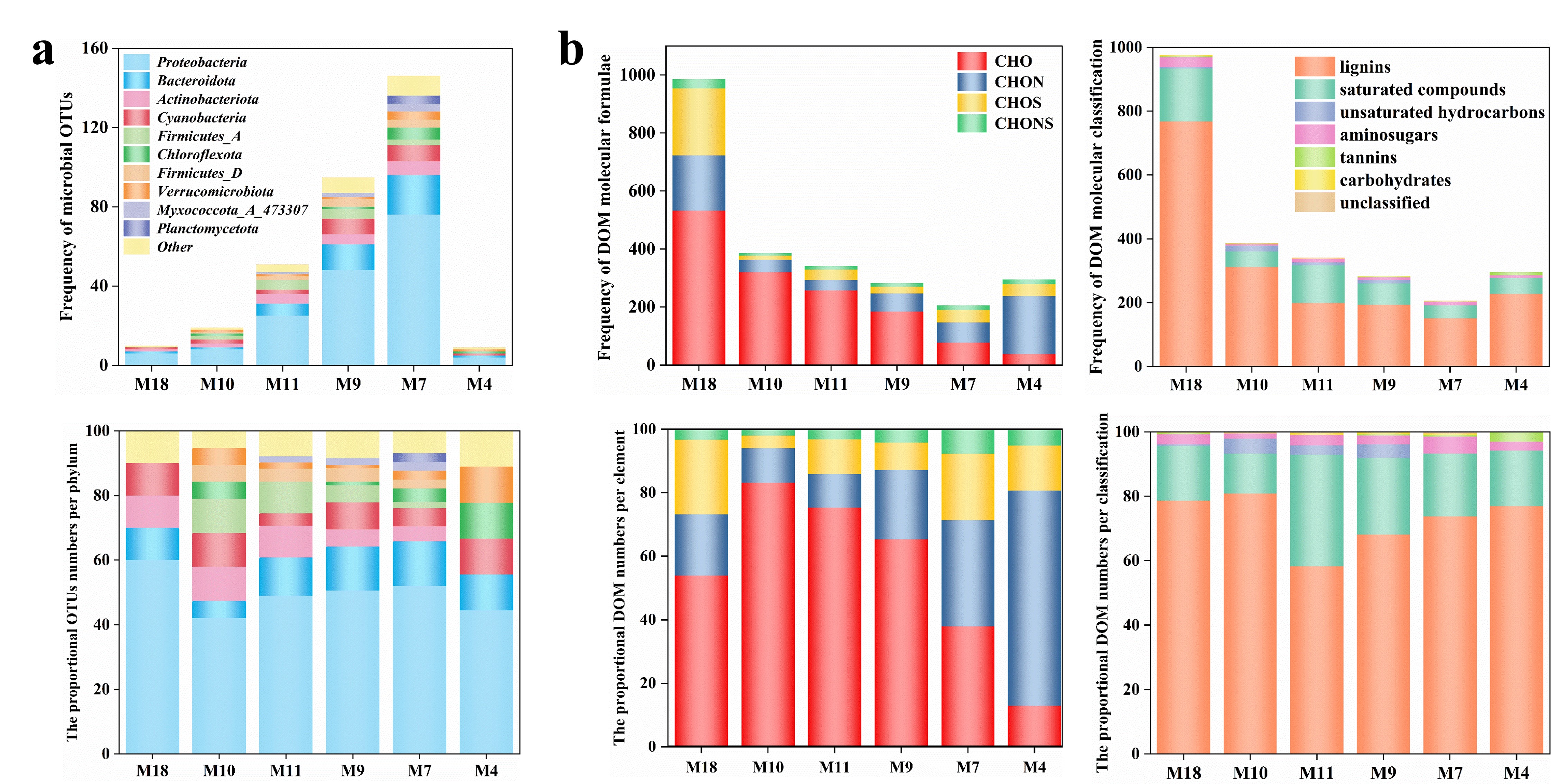
**

**Figure S22** Different modules contained distinct taxa collection (a) and DOM molecule composition (b) in the bacteria-DOM network. M18 was the abbreviation of module 18 in bacteria-DOM network, and similar expressions were also applied to other modules.

**
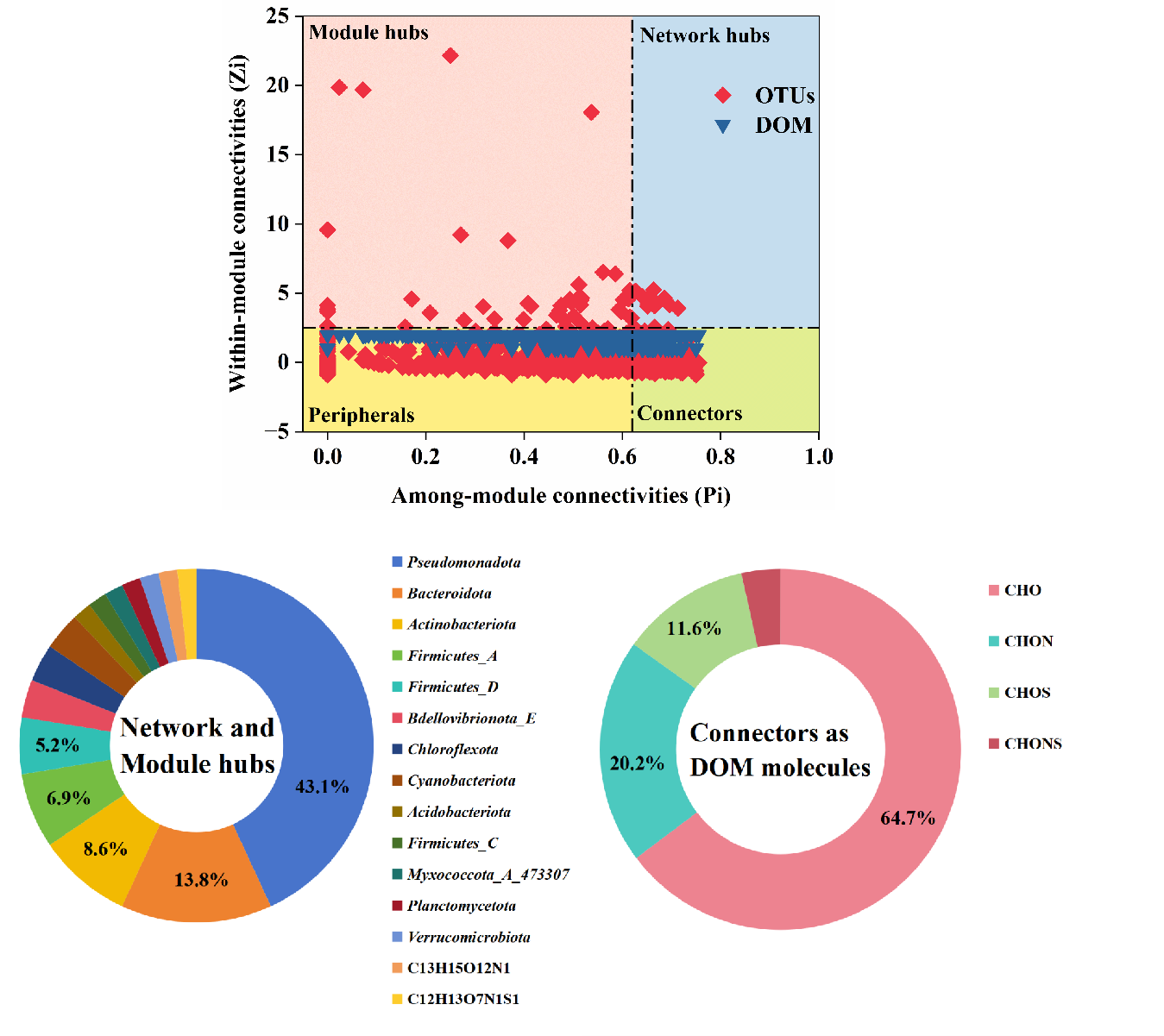
**

**Figure S23** Roles of the bacterial OTUs and DOM molecules identified as the network nodes. Only calculated values of some hubs and connectors were labeled for clarity.

**
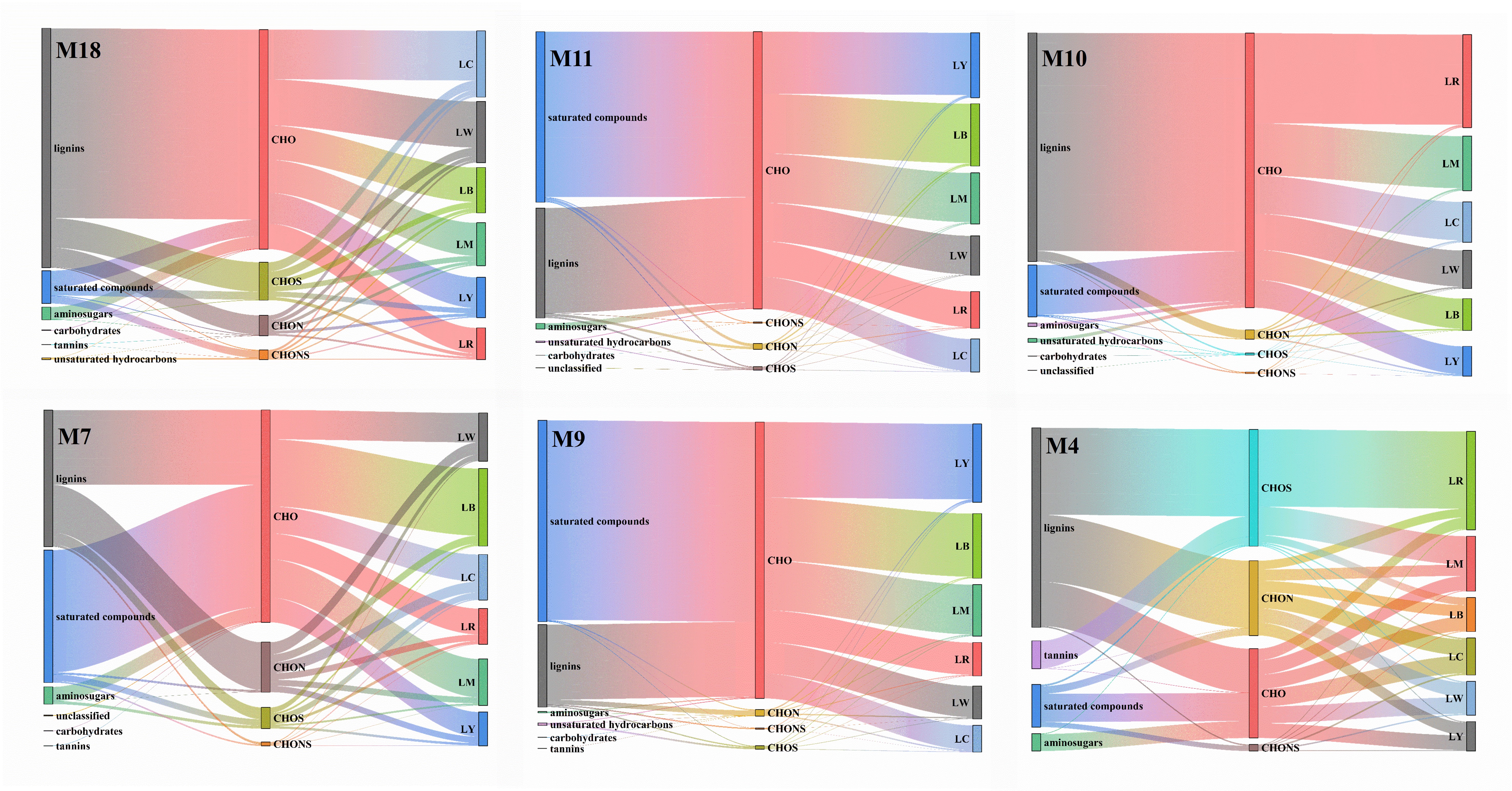
**

**Figure S24** The relative abundance of DOM metabolites related to different ecological clusters under different treatments, according to the classification and element composition of molecules. LC represented as the normal lake water; LB, LW, LR and LY respectively expressed as the lake water added DOM filtration derived from black, white, red and yellow PE; LM was set as a combined treatment, converging four collected PE-derived DOM filtration. M18 was the abbreviation of module 18 in bacteria-DOM network, and similar expressions were also applied to other modules.**Supplementary Tables**

**Table S1.** Detailed description of the four fluorescence components of PE-derived DOM identified by PARAFAC.

| **Components** | **Ex (nm)** | **Em (nm)** | **Description** |
| --- | --- | --- | --- |
| C1 | 295 | 405 | Humic-like substances |
| C2 | 270 (295) | 360 | Protein/Phenol-like substances |
| C3 | 270 | 310 | Protein/Phenol-like substances |
| C4 | 320 | 450 | Humic-like substances |

**Table S2.** An overview of the FT-ICR MS average molecular parameters for the detected DOM in the current study.

| **Samples** | **M/Z_wa_** | **O/C_wa_** | **H/C_wa_** | **AI_Modwa_** | **DBE_wa_** | | **NOSC_wa_** |
| --- | --- | --- | --- | --- | --- | --- | --- |
| **PE-derived DOM** |  |  |  |  |  | |  |
| **White_PE** | 268.22 | 0.29 | 1.60 | 0.15 | 4.15 | | -0.99 |
| **Black_PE** | 308.83 | 0.30 | 1.55 | 0.16 | 4.90 | | -0.95 |
| **Yellow_PE** | 308.81 | 0.31 | 1.56 | 0.15 | 4.71 | | -0.92 |
| **Red_PE** | 344.95 | 0.41 | 1.14 | 0.30 | 8.28 | | -0.18 |
| **Day 0** |  |  |  |  |  | |  |
| **LC** | 341.13 | 0.33 | 1.40 | 0.23 | 6.57 | | -0.65 |
| **LB** | 315.12 | 0.31 | 1.46 | 0.20 | 5.69 | | -0.77 |
| **LW** | 334.97 | 0.33 | 1.43 | 0.22 | 6.28 | | -0.70 |
| **LR** | 344.22 | 0.30 | 1.37 | 0.26 | 7.02 | | -0.71 |
| **LY** | 310.67 | 0.32 | 1.50 | 0.19 | 5.33 | | -0.79 |
| **LM** | 321.29 | 0.33 | 1.43 | 0.22 | 6.03 | | -0.71 |
| **Day 7** |  |  |  |  |  | |  |
| **LC** | 349.61 | 0.35 | 1.37 | 0.24 | 6.83 | | -0.60 |
| **LB** | 320.17 | 0.32 | 1.45 | 0.21 | 5.82 | | -0.74 |
| **LW** | 345.84 | 0.35 | 1.40 | 0.23 | 6.62 | | -0.63 |
| **LR** | 335.78 | 0.35 | 1.31 | 0.26 | 7.06 | | -0.52 |
| **LY** | 314.18 | 0.32 | 1.47 | 0.20 | 5.64 | | -0.76 |
| **LM** | 323.68 | 0.34 | 1.39 | 0.23 | 6.31 | | -0.64 |
| Legend: wa, weighted-average. | | | | | |  | |

**Table S3.** General chemical properties of lake water samples.

| **Samples** | DOC  (mg/L) | DON  (mg/L) | PO_4_^3-^  (mg/L) | NO_2_^-^  (mg/L) | NO_3_^-^  (mg/L) | NH_4_^+^  (mg/L) |
| --- | --- | --- | --- | --- | --- | --- |
| **On site** | 4.77±0.01 | 3.24±0.02 | 0.04±0.00 | 0.16±0.00 | 0.77±0.00 | 1.97±0.01 |
| **Day 0** |  |  |  |  |  |  |
| LC | 4.50±0.18 | 2.98±0.01 | 0.06±0.01 | 0.12±0.00 | 0.54±0.00 | 1.73±0.00 |
| LB | 5.89±0.04 | 3.07±0.04 | 0.07±0.04 | 0.13±0.00 | 0.68±0.00 | 1.77±0.00 |
| LW | 5.85±0.08 | 3.20±0.08 | 0.08±0.01 | 0.13±0.00 | 0.53±0.00 | 1.89±0.00 |
| LR | 6.70±0.17 | 2.96±0.17 | 0.07±0.02 | 0.13±0.00 | 0.71±0.00 | 1.97±0.00 |
| LY | 5.79±0.07 | 3.11±0.07 | 0.07±0.02 | 0.13±0.00 | 0.57±0.00 | 1.85±0.00 |
| LM | 5.74±0.09 | 3.11±0.09 | 0.07±0.01 | 0.13±0.00 | 0.62±0.00 | 1.86±0.01 |
| **Day 7** |  |  |  |  |  |  |
| LC | 4.31±0.19 | 4.34±0.37 | 0.05±0.02 | 0.12±0.00 | 0.49±0.00 | 1.91±0.01 |
| LB | 5.25±0.35 | 4.54±0.04 | 0.02±0.02 | 0.12±0.00 | 0.61±0.00 | 1.90±0.00 |
| LW | 4.92±0.30 | 3.82±0.39 | 0.02±0.02 | 0.12±0.00 | 0.49±0.00 | 1.91±0.00 |
| LR | 5.51±0.70 | 4.65±0.16 | 0.03±0.03 | 0.12±0.00 | 0.54±0.00 | 1.90±0.00 |
| LY | 4.70±0.66 | 5.21±0.06 | 0.04±0.01 | 0.12±0.00 | 0.50±0.00 | 1.93±0.00 |
| LM | 4.79±0.55 | 4.82±0.03 | 0.03±0.02 | 0.12±0.00 | 0.52±0.00 | 1.82±0.00 |

**Table S4.** Detailed description of the five fluorescence components of DOM from lake water samples identified by PARAFAC.

| **Components** | **Ex (nm)** | **Em (nm)** | **Description** |
| --- | --- | --- | --- |
| C1 | ＜250 (330) | 425 | Terrestrial humic-like substances |
| C2 | 295 | 410 | Microbial humic-like substances |
| C3 | 290 | 365 | Microbial tryptophane-like substances |
| C4 | 270 | 330 (520) | Microbial tryptophan-like substances; Fulvic-like substances |
| C5 | 330 (365) | 470 (520) | Unknown mixed humic-like substances |

**References**

1. Murphy KR, Butler KD, Spencer RG *et al.* Measurement of dissolved organic matter fluorescence in aquatic environments: An interlaboratory comparison. *Environ Sci Technol.* **2010**, 44, 9405–12.

2. Lawaetz AJ, Stedmon CA. Fluorescence intensity calibration using the raman scatter peak of water. *Appl Spectrosc.* **2009**, 63, 936–40.

3. Stedmon CA, Bro R. Characterizing dissolved organic matter fluorescence with parallel factor analysis: A tutorial. *Limnol Oceanogr Methods.* **2008**, 6, 572–79.

4. Murphy KR, Stedmon CA, Graeber D *et al.* Fluorescence spectroscopy and multi-way techniques. Parafac. *Anal Methods.* **2013**, 5, 6557–66.

5. Sun Y, Li X, Li X *et al.* Deciphering the fingerprint of dissolved organic matter in the soil amended with biodegradable and conventional microplastics based on optical and molecular signatures. *Environ Sci Technol.* **2022**, 56, 15746–59.

6. Lee YK, Murphy KR, Hur J. Fluorescence signatures of dissolved organic matter leached from microplastics: Polymers and additives. *Environ Sci Technol.* **2020**, 54, 11905–14.

7. An S, Mao Z, Chen M *et al.* Sunlight irradiation promotes both the chemodiversity of terrestrial dom and the biodiversity of bacterial community in a subalpine lake. *Environ Res.* **2023**, 227, 115823.

8. Eder A, Weigelhofer G, Pucher M *et al.* Pathways and composition of dissolved organic carbon in a small agricultural catchment during base flow conditions. *Ecohydrol Hydrobiol.* **2022**, 22, 96–112.

9. Graeber D, Gelbrecht J, Pusch MT *et al.* Agriculture has changed the amount and composition of dissolved organic matter in central european headwater streams. *Sci Total Environ.* **2012**, 438, 435–46.

10. Williams CJ, Yamashita Y, Wilson HF *et al.* Unraveling the role of land use and microbial activity in shaping dissolved organic matter characteristics in stream ecosystems. *Limnol Oceanogr.* **2010**, 55, 1159–71.

11. Murphy KR, Stedmon CA, Wenig P *et al.* Openfluor– an online spectral library of auto-fluorescence by organic compounds in the environment. *Anal Methods.* **2014**, 6, 658–61.

12. Koch BP, Dittmar T. From mass to structure: An aromaticity index for high-resolution mass data of natural organic matter. *Rapid Commun Mass Spectrom.* **2006**, 20, 926–32.

13. Kellerman AM, Kothawala DN, Dittmar T *et al.* Persistence of dissolved organic matter in lakes related to its molecular characteristics. *Nat Geosci.* **2015**, 8, 454–57.

14. Zhang B, Shan C, Wang S *et al.* Unveiling the transformation of dissolved organic matter during ozonation of municipal secondary effluent based on ft-icr-ms and spectral analysis. *Water Res.* **2021**, 188, 116484.
